# Revisiting Coulombic Efficiency Paradigm: Electrolyte Additive Design for Lithium Metal Batteries

**DOI:** 10.1002/anie.9595705

**Published:** 2026-07-31

**Authors:** Qixin Wang, Jiaxun Yang, Pu Li, Kun Zhao, Xinxin Zhang, Bowen Zheng, Qiangfu Sun, Zhongyang Zhang, Wenfang Feng, Javier Carrasco, Hailong Yu, Gebrekidan Gebresilassie Eshetu, Egbert Figgemeier, Michel Armand, Xuejie Huang, Zhibin Zhou, Heng Zhang

**Affiliations:** ^1^ Key Laboratory of Material Chemistry for Energy Conversion and Storage (Ministry of Education), School of Chemistry and Chemical Engineering Huazhong University of Science and Technology Wuhan China; ^2^ Chinese Academy of Sciences Beijing China; ^3^ Centre for Cooperative Research on Alternative Energies (CIC energiGUNE) Basque Research and Technology Alliance (BRTA) Vitoria‐Gasteiz Spain; ^4^ IKERBASQUE Ikerbasque, Basque Foundation for Science Bilbao Spain; ^5^ Institute of Power Electronics and Electric Drives (ISEA), Center for Ageing Reliability and Lifetime Prediction of Electrochemical and Power Electronic Systems (CARL) RWTH Aachen University Aachen Germany; ^6^ Department of Materials Science and Engineering, Mekelle Institute of Technology Mekelle University Mekelle Ethiopia; ^7^ Helmholtz Institute Münster (HIMS), Institute of Energy Materials and Devices (IMD 4) Forschungszentrum Jülich GmbH Münster Germany; ^8^ Jülich Aachen Research Alliance JARA‐Energy Jülich Germany

**Keywords:** coulombic efficiency, decomposition mechanism, electrolyte additive, nonafluorobutanesulfonyl fluoride, SEI formation

## Abstract

Small‐dose electrolyte additives are widely used to enhance battery performance, yet rational additive selection for the stabilization of lithium metal (Li°) electrodes remains challenging. Coulombic efficiency (CE) measurements in asymmetric Li°||Cu° cells serve as critical evaluative metrics, but the resulting CE values often deviate from expectations based on additive structure alone. Herein, we delve into the design of fluorinated electrolyte additives by combining statistical analysis of Li°||Cu° cycling data with compositional characterization as well as atomistic and chemical simulations. Our results demonstrate that nonafluorobutanesulfonyl fluoride, as an electrolyte additive, induces only a marginal effect on the Aurbach CE values during short‐term cycling tests of Li°||Cu° cells. Intriguingly, during extended‐cycling, statistical analysis reveals that the same additive exhibits divergent effects in electrolyte families based on bis(trifluoromethanesulfonyl)imide (TFSI^−^) and bis(fluorosulfonyl)imide (FSI^−^) anions. These differences arise from the interplay between the film‐forming chemistry of the fluorinated additive and the active involvement of TFSI^−^ and FSI^−^ anions, which collectively modulate the chemical and electrochemical features of the resulting solid‐electrolyte interphases (SEI) on Li°. This work elucidates how additive functionality translates into interphase chemistry and provides a statistically robust framework for screening electrolyte additives for practical lithium metal batteries.

## Graphical abstract

Coulombic efficiency (CE) is a key metric for screening advanced electrolyte formulations for rechargeable batteries. Combined with interphasial characterizations, statistical analyses of CE data uncover the synergistic interaction between fluorinated additives and salt anions, enabling more precise correlations of chemical functionalities with electrochemical performances.

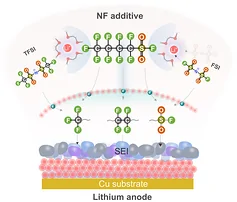

## 1 Introduction

Coulombic efficiency (CE) is a critical parameter that dictates capacity retention, long‐term lifespan, and energy efficiency, a factor that determines the overall cost of rechargeable battery technologies [1, 2, 3]. In graphite negative electrode‐based commercial lithium‐ion batteries (LIBs), the lithium‐ion inventory is limited by the amount of lithium supplied from the positive (cathode) electrode, and irreversible consumption of active lithium sources consequently leads to substantial losses in capacity and shortened lifetime of LIB cells [1, 4] (Figure 1A). With an average CE of 99.00%, graphite‐based LIB cells lose up to 60% of their initial capacity after only 100 cycles. To meet electric vehicle (EV) requirements of 1,000 cycles while limiting capacity loss to 20%, the average CE must be maintained above an exceptionally high value of 99.99% (Figure 1B). Moreover, increasing the CE by only 0.01% can roughly double the cycle life of LIB cells, highlighting the critical importance of CE when evaluating batteries for practical applications. The historical evolution of LIB technology has clearly demonstrated that the use of electrolyte additives has been a key strategy for achieving CE values greater than 99.99% [2, 5]. For example, vinylene carbonate (VC) has been widely proven since the 1990s to effectively promote the formation of a stable and elastic solid‐electrolyte‐interphase (SEI) layer on the graphite negative electrode [6, 7]. As a result, it has become a benchmark additive for the development of new electrolytes in LIBs. Furthermore, the incorporation of other functional additives has drastically extended the cycling lifespan of LIBs from a few hundred cycles to tens of thousands of cycles, as evidenced by the contemporary graphite||LiFePO_4_ batteries [2, 5].

**FIGURE 1:**
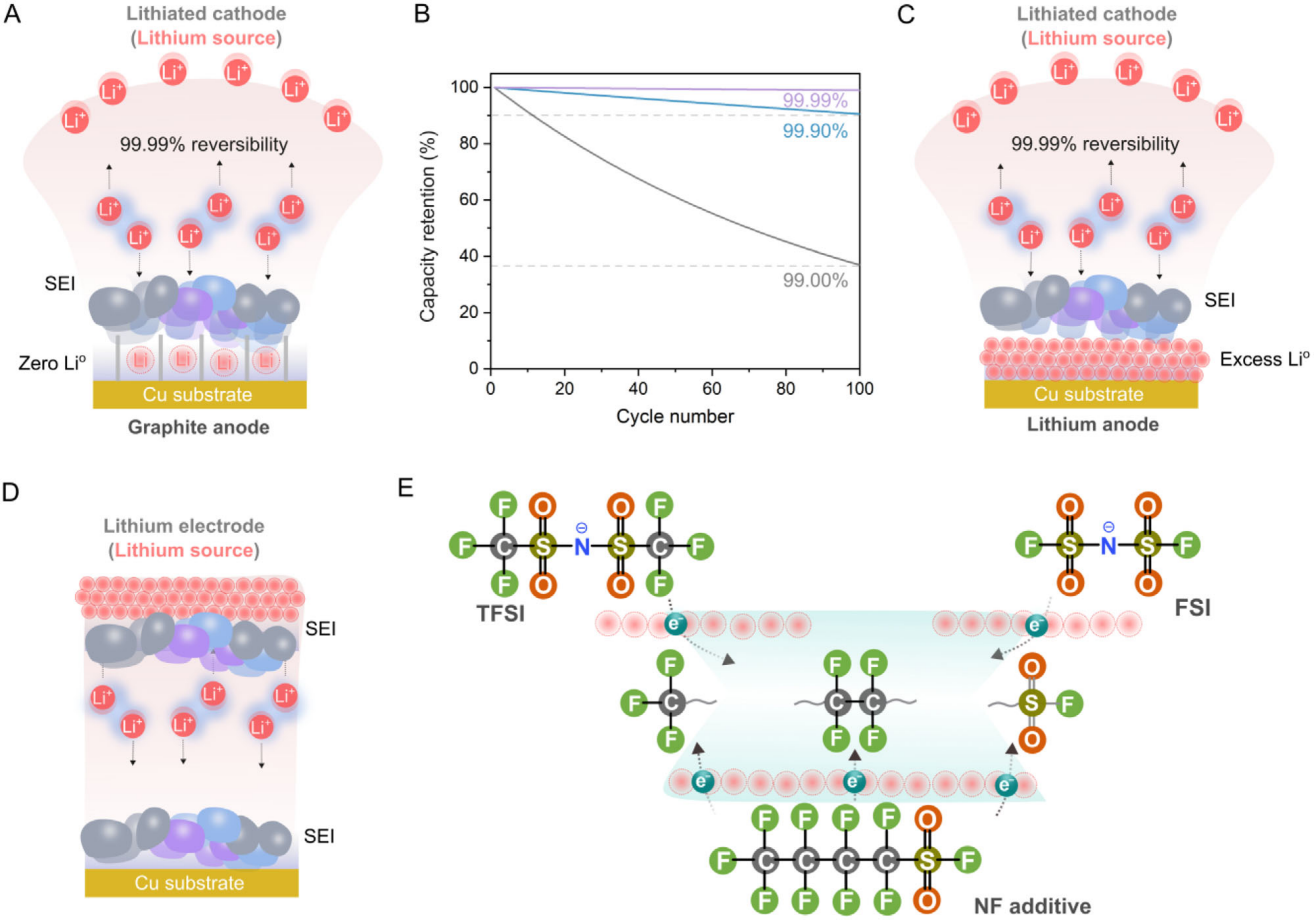
Conceptual design of NF‐added electrolytes. (A) Distribution of electroactive lithium in the graphite‐based cells. (B) Dependence of cycle life on the average Coulombic efficiency. (C) Distribution of electroactive lithium in lithium metal‐based cells. (D) Cell configuration of lithium||copper asymmetric cells. (E) Chemical structures the NF additive and TFSI^−^/FSI^−^ salt anion and their linkage with SEI formation pathways.

Leveraging the exceptionally high theoretical specific capacity (3,860 mAh g^−1^) of metallic lithium (Li°) and its alloys (e.g., lithium–silicon/Li‐Si alloy, lithium–tin/Li‐Sn alloy, lithium–aluminum/Li‐Al alloy, and lithium–indium/Li‐In alloy), rechargeable lithium metal batteries (LMBs) can deliver significantly higher specific energy densities, far exceeding the prevailing state‐of‐the‐art graphite‐based LIBs [8]. Consequently, LMBs are widely regarded as promising next‐generation energy storage systems for emerging applications such as electric vehicles, low‐altitude transportation, and robotics [9].

Analogous to the graphite negative electrode in LIB technology, the formation of a chemo‐mechanically stable SEI layer on the Li° negative electrode is even more critical for maintaining high cycling efficiency and cycling stability of rechargeable LMBs [10, 11, 12] (Figure 1C). Fluoroethylene carbonate (FEC) and other fluorinated additives have received considerable attention because of their strong ability to generate lithium fluoride (LiF) as a key SEI building component, thereby increasing the mechanical robustness and reducing electron leakage of the resulting SEI layer [13, 14, 15]. These fluorinated electrolyte additives, originally developed in the context of LIB electrolyte design and optimization, have therefore been widely adopted in LMB systems. In particular, FEC is able to stabilize Li° and its Li‐alloy electrodes through ring‐opening polymerization at relatively high potentials (> 1.0 V vs. Li/Li^+^), occurring prior to the reductive decomposition of electrolyte solvents and salt anions, as well as the onset of electrochemical lithium plating [14]. In the same vein, fluorinated additives containing fluorosulfonyl (FSO_2_–) groups have been identified as effective SEI‐building precursors, owing to their high tendency of film‐forming ability that enables the regulation of both the spatial structure and chemical composition of SEI layers. Bis(fluorosulfonyl)imide anion (FSI^−^) is an archetypical member of this class of electrolyte additives [16, 17, 18]. The presence of FSO_2_– moiety, featuring a relatively weak S─F bond, facilitates the formation of inorganic sulfate and fluoride species on metal electrodes. These decomposition products contribute to the construction of a mechanically robust SEI layer, thereby improving interfacial stability [17, 19]. Similar beneficial effects have also been reported for other fluorosulfonyl‐containing compounds, such as *N*, *N*‐dimethylaminosulfonyl fluoride (MeFSA), and *N, N*‐diethyl sulfamoylfluoride (EtFSA) [20].

In recent years, asymmetric lithium||copper (Li°||Cu°, Figure 1D) cells have been widely employed to assess the performance of electrolyte additives by evaluating the average CE over several cycles, commonly referred to as the Aurbach tests [9, 21, 22, 23, 24]. In stark contrast, Li° and Li‐alloy electrode materials used in rechargeable LMBs act as lithium reservoirs, supplying electrochemically active lithium ions that can partially compensate for irreversible lithium losses. This excess lithium masks the effects of parasitic reactions, making it more challenging to rapidly and reliably identify effective electrolyte additives [25, 26, 27] (Supplementary Note #1, Figure 1C). These limitations underscore the critical importance of CE and the need for its precise evaluation. High CE values are often considered a key criterion for screening electrolyte additives prior to further evaluation in cells paired with cathode materials [24, 28]. However, the chemical nature of copper electrodes differs substantially from that of Li° and Li‐alloy electrodes, which can introduce uncertainties and potential discrepancies in the evaluation of electrolyte additive performance [1, 23, 29].

In this work, we delve into the design of fluorinated electrolyte additives for rechargeable LMBs, with a particular focus on the statistical analysis of Li°||Cu° data and their correlation with chemical identities derived from compositional characterization and chemical simulations. Parent electrolytes based on lithium bis(fluorosulfonyl)imide (LiFSI) and lithium bis(trifluoromethanesulfonyl)imide (LiTFSI) were selected due to the distinct SEI‐forming abilities of FSI^−^ versus TFSI^−^ anions. Nonafluorobutanesulfonyl fluoride (NF) has been proposed as an electrolyte additive for rechargeable LMBs, owing to its film‐forming capability arising from the FSO_2_– functional group and its surfactant‐like properties stemming from the C_4_F_9_– moieties [30]. Herein, NF is deliberately employed as a model fluorinated additive because of this dual chemical functionality as well as its minimal impact on Li°||Cu° cycling performance. These characteristics make it particularly suitable for demonstrating how statistical approaches can be applied for the rigorous evaluation of electrolyte additives in future research studies (Figure 1E). By combining compositional characterization with chemical simulations, we elucidate the competitive decomposition pathways between the NF additive and FSI^−^/TFSI^−^ salt anions, as well as their impacts on the chemical and electrochemical properties of Li° interphases. These findings provide deeper mechanstic insights into the rational design of advanced electrolyte additives, paving the way for the development of high‐performance  rechargeable batteries.

## 2 Results and Discussion

### 2.1 Thermal and Transport Properties of the NF‐containing Electrolytes

For the parent electrolytes, that is, without NF additive, the conducting salts (e.g., LiTFSI and LiFSI) were dissolved in 1,2‐dimethoxyethane (DME) up to a molarity of 1.0 mol L^−1^. The amount of NF additive was quantified by its mass fraction vs. parent electrolytes. Table 1 presents the fundamental properties of the as‐prepared electrolytes [e.g., ionic conductivity (*σ*), Crystallization transition temperature (*T*_c_), and melting transition temperature (*T*_m_)], in which the acronyms denote salt identity and weight percentage of NF additive (e.g., LTF‑3N implies 1.0 M LiTFSI‑DME + 3 wt% NF). Figure 2A shows the physical appearance of the parent electrolytes before and after the addition of NF additives. In both LiTFSI and LiFSI‐based electrolytes, a small portion of NF additive tends to be miscible without clear phase separation; yet, exclusion of NF phase occurs beyond these limiting values (e.g., 15 wt% for LiTFSI‐based electrolyte and 7 wt% for LiFSI‐based electrolyte; Figure 2A) [31]. Accordingly, three NF additive contents (1, 3, and 5 wt%) were designated in subsequent investigations.

**TABLE 1:** Electrolyte compositions and fundamental physical properties of LiTFSI‐ and LiFSI‐based electrolytes.

Acronym	Electrolyte composition^a^	*σ* (mS cm^−1^)^b^	*T*_c_ (°C)^c^	*T*_m_ (°C)^d^
LTF‐0N	1.0 M LiTFSI/DME	14.27 ± 0.017	/	−86, −68
LTF‐1N	1.0 M LiTFSI/DME + 1 wt% NF	14.05 ± 0.030	/	−85, −69
LTF‐3N	1.0 M LiTFSI/DME + 3 wt% NF	13.77 ± 0.029	/	−86, −69
LTF‐5N	1.0 M LiTFSI/DME + 5 wt% NF	13.48 ± 0.006	/	−86, −69
LF‐0N	1.0 M LiFSI/DME	17.60 ± 0.021	−116, −106, −85	−68
LF‐1N	1.0 M LiFSI/DME + 1 wt% NF	17.60 ± 0.021	−112, −103, −85	−69
LF‐3N	1.0 M LiFSI/DME + 3 wt% NF	17.23 ± 0.001	−114, −106, −85	−69
LF‐5N	1.0 M LiFSI/DME + 5 wt% NF	16.80 ± 0.004	−111, −106, −85	−69

^a^ The weight percentage of NF is calculated based on the mass of each individual reference electrolyte.

^b^ Ionic conductivity was measured at 25 °C, and all data are presented as the mean ± standard deviation (SD) from three independent measurements.

^c^ The average temperature of crystallization transition determined by DSC under heating scan.

^d^ The average temperature of the melting transition determined by DSC under heating scan.

**FIGURE 2:**
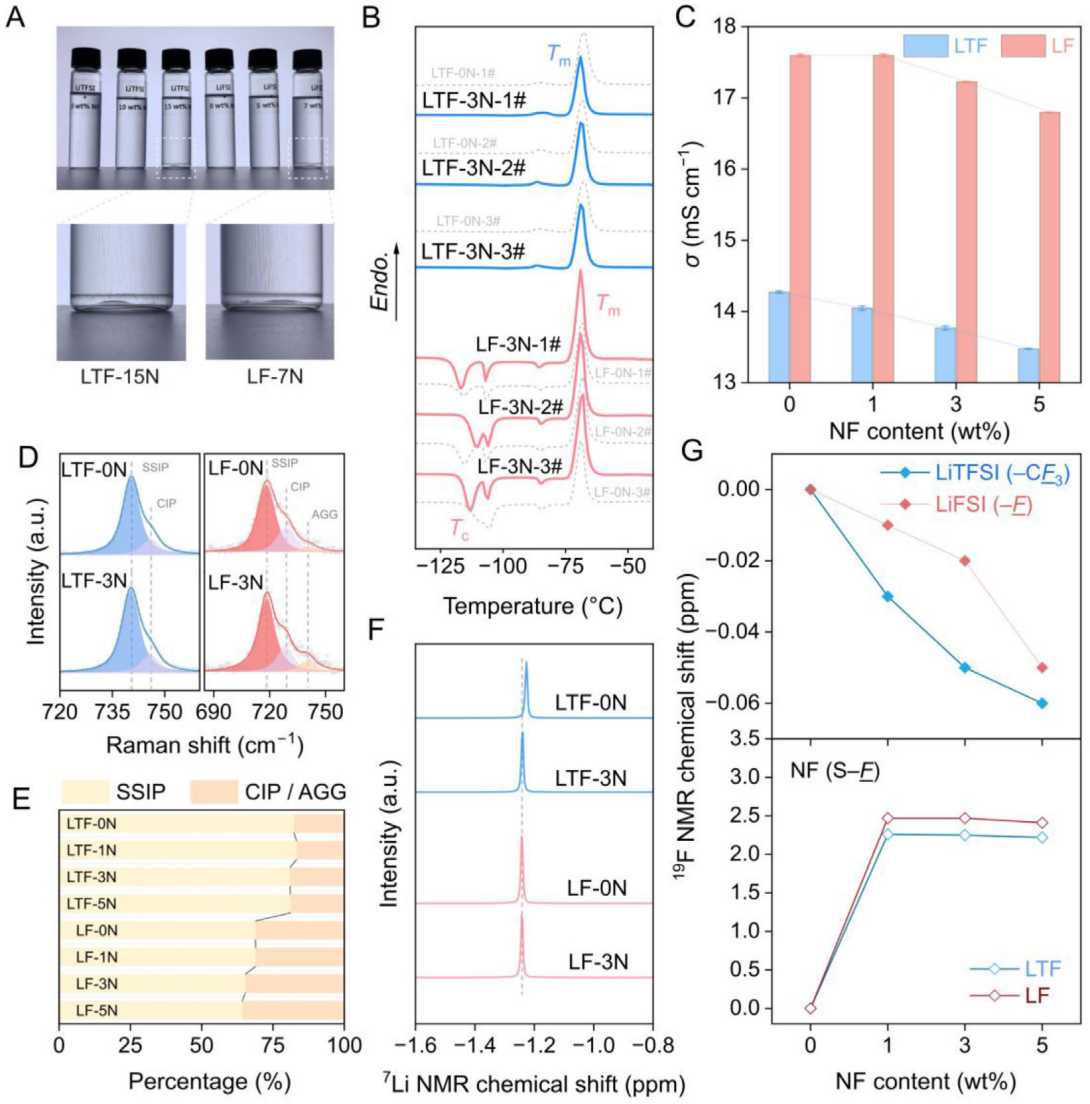
Thermal and transport properties of the NF‐containing electrolytes. (A) Physical appearance of the LiTFSI‐ and LiFSI‐based electrolytes with different amounts of NF additives. Phase separation (zoomed‐in figures) is noticed for the electrolytes with high NF content [e.g., 15 wt% for LiTFSI system (LTF‐15N) and 7 wt% for LiFSI system (LF‐7N)]. (B), (C) DSC curves (B) and ionic conductivities at 25 °C (C) of the LiTFSI‐ and LiFSI‐based electrolytes with different amounts of NF additives. (D) The Raman spectra of LiTFSI‐ and LiFSI‐based electrolytes in the selected region. (E) Percentages of solvent‐separated ion pair (SSIP) contact ion pair (CIP) and aggregate (AGG) species (normalized to 100%) in different electrolytes. (F) ^7^Li NMR spectra of LiTFSI‐ and LiFSI‐based electrolytes. (G) Evolution of chemical shifts extracted from the ^19^F NMR spectra of LiTFSI‐ and LiFSI‐based electrolytes with different amounts of NF additives.

Figure 2B shows the differential scanning calorimetry (DSC) heating curves (selected temperature range) of three parallel samples of LiTFSI‑ and LiFSI‑based electrolytes, including the parent and NF‐containing electrolytes (full‐range DSC curves in Figure S1). Without NF, the LiTFSI‐based sample (LTF‑0N) exhibits two consecutive endothermic peaks upon heating at approximately −86 °C and −68 °C, corresponding to melting of the LiTFSI‑DME complex and free DME, respectively [32, 33]. In contrast, for the LiFSI‐based electrolyte (LF‑0N), three exothermic peaks are observed upon heating at about −116 °C, −106 °C, and −85 °C, with slight variations among the three samples attributed to differences in thermal history [34]. These exothermic transitions are followed by an endothermic peak at approximately −68 °C, assigned to DME melting. The distinct thermal behavior highlights the critical role of the salt anion in that the smaller FSI^−^ anion favors low‑melting salt‑DME complexes [33], thereby suppressing crystallization during cooling. Instead, crystallization occurs gradually at low temperatures (lower than −80 °C) during subsequent heating (*aka*., cold crystallization) [33, 35]. The addition of NF has only a marginal impact on the crystallization and melting behavior of either electrolyte.

Figure 2C presents the average ionic conductivity values (with error bars) obtained from three parallel measurements at 25 °C for both LiTFSI‐ and LiFSI‐based electrolytes as a function of NF content. Without NF, the LiTFSI‐based electrolyte exhibits lower conductivity than the LiFSI‐based counterpart [36, 37] (e.g., 14.27 ± 0.017 mS cm^−1^ for LTF‐0N vs. 17.60 ± 0.021 mS cm^−1^ for LF‐0N), a trend maintained after NF addition due to the smaller size of the FSI^−^ anion (95 Å^3^ vs. 147 Å^3^ for TFSI^−^) [17, 37]. At 5 wt% NF, conductivity drops by 0.8 mS cm^−1^ in both systems, corresponding to a 5.5% and 4.4% decrease for LTF‐5N and LF‐5N, respectively, indicating a slightly stronger relative impact on the LiTFSI system. Notably, at 1 wt% NF, the LiTFSI system exhibits a pronounced conductivity drop (1.5%), whereas the LiFSI system remains unchanged. Linear fitting of conductivity versus NF content from 1 to 5 wt% yields a comparable linear decline in both systems (Figure S2). Overall, even trace amounts of NF (< 1 wt%) influence ion transport in the LiTFSI system, while the LiFSI system is barely insensitive below 1 wt% NF. However, above 1 wt%, both systems exhibit a similar declining trend.

To investigate the coordination environment of ionic species in the two electrolyte systems, Raman and NMR spectra were collected, and the source spectral profiles are presented in Figure S3. Figure 2D shows the Raman spectra in the S–N–S stretching vibration region (i.e., 680 to 760 cm^−1^). For the LiTFSI‐based electrolyte, the peaks at approximately 740  and 746  cm^−1^ are assigned to the solvent‐separated ion pair (SSIP) and contact ion pair (CIP), respectively [36, 38]. As the NF additive gradually increases to 5 wt%, the peak positions do not change significantly. Deconvolution (Figure 2E) shows that CIP species account for only 20% of total solvated ions, and this fraction remains relatively stable with varying NF content (e.g., 17–18%). This indicates TFSI^−^ anions are largely excluded from the primary solvation sheath of Li^+^ [36], and the addition of NF has only a minor influence on the solvation structure of TFSI^−^ anions. For the LiFSI‐based electrolyte, the characteristic Raman peaks for SSIP, CIP, and aggregate (AGG) [36] appear at approximately 718, 728, and 740 cm^−1^, respectively. Compared with LiTFSI‐based electrolyte, the SSIP fraction is markedly lower (e.g., 69% for LF‐0N vs. 82% for LTF‐0N) [38]. Upon introducing the NF additive, the peak positions of the three characteristic structures and the proportion of SSIP do not change significantly. These results demonstrate that in the bulk phase, FSI^−^ anions preferentially coordinate directly with Li^+^, a configuration that significantly suppresses the degree of crystallization at low temperatures. Additional Raman analyses in other relevant regions are provided in the Supporting Information (Tables S1 and S2), and the corresponding results, aligning with those obtained from the 680–760 cm^−1^ region, further confirming that NF does not significantly alter the solvation structure of either LiTFSI or LiFSI electrolytes.

The chemical shifts of NMR‐sensitive nuclei (i.e., ^1^H, ^7^Li and ^19^F) provide insights into the solution‐state chemical environments of electrolyte species [39]. ^1^H NMR spectra (Figure S3) show that the methyl (C **H**_3_O─) and methylene (─C **H**_2_─) proton signals of DME in both LiTFSI‐ and LiFSI‐based electrolytes shift upfield relative to neat DME (e.g., methylene hydrogen: 3.84 ppm for LTF‐0N vs. 3.97 ppm for DME), indicating that Li^+^ coordination alters the electron density of DME [40]. ^7^Li NMR (Figure 2F) reveals negligible changes upon the incorporation of NF additive (e.g., −1.23 ppm for LTF‐0N vs. −1.24 ppm for LTF‐3N), which suggests that NF does not directly participate in the primary coordination shell of Li^+^ ions. This is consistent with the observed unchanged ^1^H NMR signals after adding NF additive into each electrolyte system (Figure S3). Intriguingly, from the perspective of salt anions, the ion‐solvent and ion‐ion interactions are somehow interfered upon addition of NF additive. Figure 2G displays the evolution of chemical shifts associated with the two salt anions and NF additive. After adding NF, the TFSI^−^ anion exhibits a more pronounced upfield shift than the FSI^−^ anion, e.g., −0.05 ppm for LTF‐3N vs. −0.02 ppm for LF‐3N. These changes clearly reflect extra solvent‐solvent and ion‐solvent interactions brought by the fluorinated NF additive. Moreover, the influence of NF on the ion‐solvent interactions differs between the two anions.

In brief, the addition of a small dosage of NF additive does not significantly affect the thermal behavior and transport properties of the bulk electrolytes, due to its chemical inertness in the solvation of cation and anionic species. Nevertheless, the weak interactions between NF and salt anions and solvent molecules may alter the solvation structure of salt anions and free solvents, thus affecting the chemical and electrochemical properties of the NF‐added electrolytes, as further analyzed in Figure 4.

### 2.2 Solvation Structures and Local Microenvironment of NF Additives

Building upon the macroscopic analyses in Figure 2, introducing trace amounts of NF additive (0–5 wt%) has only a minor effect on the intrinsic solvation behavior of lithium salts in DME‐based electrolytes. To further resolve into the inner solvation sheath of ionic species and the atomic‐scale distribution of NF molecules, molecular dynamics (MD) simulations coupled with density functional theory (DFT) calculations were performed, with a focus on the optimized NF content of 3 wt% (Figure 3).

**FIGURE 3:**
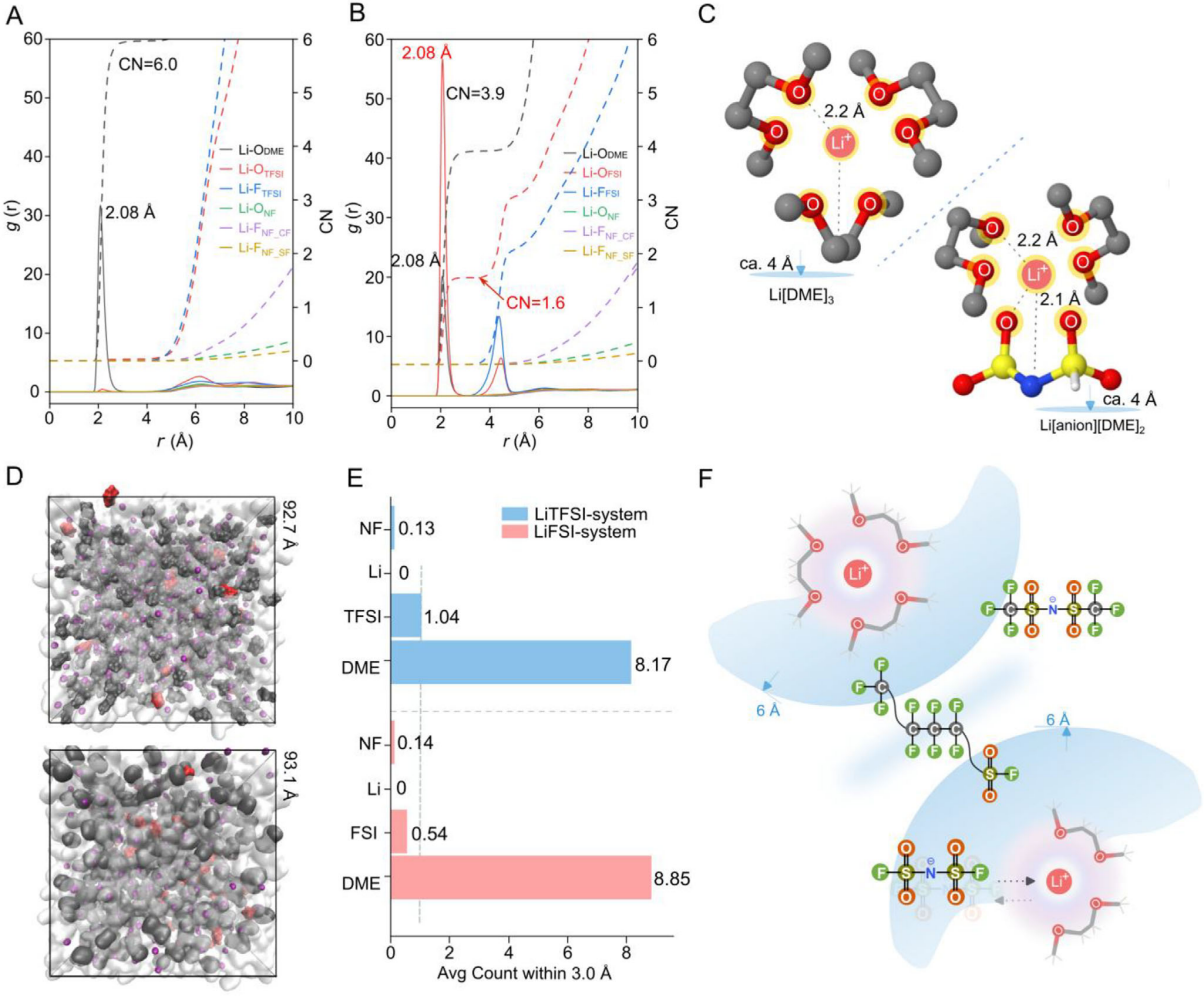
MD simulations and DFT calculations of LiTFSI‐ and LiFSI‐based electrolytes containing 3 wt% NF additive. (A), (B) Radial distribution function (RDF) and coordination number (CN) profiles of the LiTFSI‐based (A) and LiFSI‐based (B) systems. (C) Optimized gas‐phase DFT structures of the solvent‐coordinated Li[DME]_3_ complex and Li[FSI][DME]_2_, as a representative of anion‐coordinated complexes. (D) Representative snapshots of the equilibrated MD simulation boxes for the LiTFSI‐based electrolyte (top, 92.7 Å × 92.7 Å × 92.7 Å) and LiFSI electrolyte (bottom, 93.1 Å × 93.1 Å × 93.1 Å); white regions denote DME solvent, black regions denote anions, purple spheres represent Li^+^ ions, and red regions denote NF additives. (E) The average count of DME solvent molecules, anions (TFSI^−^ or FSI^−^), NF additive molecules, and Li^+^ ions within a 3.0 Å radial distance from an individual NF molecule in the LiTFSI‐ and LiFSI‐based systems. (F) Schematic illustration of the local solvation environments and spatial organization surrounding NF additives in the bulk electrolytes.

The radial distribution function (RDF) and coordination number (CN) profiles describing the Li^+^ solvation environment are shown in Figure 3A. For the 3 wt% NF ‐containing LiTFSI‐based electrolyte, the Li‐O_DME_ RDF exhibits a sharp, intense peak at approximately 2.08 Å, corresponding to a high CN of 6.0. By contrast, the CN for Li‐O_TFSI_ is essentially zero, while no contribution from NF is observed, with CN values for Li‐O_NF_ and Li‐F_NF_ remaining also zero. These results indicate that the primary Li^+^ solvation shell is almost entirely occupied by DME molecules, whereas bulky TFSI^−^ anions and NF additives are effectively excluded from the inner solvation sheath. Analysis of the specific Li^+^ inner‐shell configurations (Figure S4) shows that approximately 98% adopt sixfold coordination between Li^+^ and DME molecules (i.e., Li[DME]_3_), characteristic of a typical SSIP. Notably, this result is nearly identical to that of the pristine, 0 wt% NF, system (Figure S5a). This observation is also consistent with previous findings [41] that, in dilute and moderate concentrations, Li^+^ cations are primarily coordinated by DME molecules. Detailed analysis of the LiTFSI‐based system with 0–5 wt% NF is provided in Supplementary Note 2#. Owing to the high abundance of DME and its strong bidentate chelating ability, TFSI^−^ anions, despite their high negative charge density, are sterically excluded from the primary Li^+^ shell due to their large molecular volume and lower relative abundance compared with the solvent. These microscopic statistical results are highly consistent with the Raman and ^7^Li NMR spectra discussed above (Figure 2D,F). Specifically, Raman deconvolution confirms that SSIP configurations strongly dominate over CIP in the LiTFSI‐based electrolyte, with an SSIP:CIP ratio of approximately 8:2. Thus, the MD trajectories effectively bridge the macroscopic spectroscopic observations and the atomic‐scale solvation structure.

In stark contrast, LiFSI‐based electrolytes exhibit a fundamentally different solvation structure. As depicted in Figure 3B, the RDF and CN analyses resolve that the local coordination environment of Li^+^ displays sharp Li‐O_DME_ and Li‐O_FSI_ coordination peaks centered at approximately 2.08 Å. On average, each Li^+^ ion is coordinated by 3.9 oxygen atoms from DME molecules and 1.6 oxygen atoms from FSI^−^, yielding a total CN of approximately 5.5. This indicates substantial participation of FSI^−^ anions in the primary solvation sheath of Li^+^. Further statistical analysis of the MD trajectories reveals that ionic species in the LiFSI‐based electrolytes majorly exist as CIPs (i.e., Li[FSI][DME]_2_, ca. 60%) or aggregates (AGG, ca. 20%), whereas SSIPs are comparatively less abundant. This behavior differs markedly from that of LiTFSI‐based electrolytes, in which SSIP‐like configurations dominate [e.g., ca. 20% (LF‐3N) vs. ca. 98% (LTF‐3N), Figure S4]. Notably, this specific solvation distribution of the LiFSI‐based electrolyte is nearly identical to the LF‐0N system (Figure S6a), consistent with the previous report by Tao Ma et al. [35]. A detailed analysis of the LiFSI‐based systems containing 0–5 wt% NF is provided in Supplementary Note 3#. Collectively, these computational insights demonstrate that FSI^−^ anions possess a much stronger propensity than TFSI^−^ anions to enter the primary Li^+^ solvation sheath. This atomistic preference is in good agreement with the qualitative trends observed in the Raman spectra (Figure 2D), where the LiFSI system exhibits a markedly higher proportion of CIP and AGG species than the LiTFSI‐based system. Interestingly, Raman spectral deconvolution further suggests that increasing the NF content gradually modulates the relative populations of these solvation motifs. Although the MD simulations and Raman spectroscopy exhibit strong qualitative consistency in capturing the distinct solvation paradigms of the two salts, a precise quantitative agreement between the MD‐derived structural statistics and the Raman‐fitted species fractions is not expected. This discrepancy is reasonable given the different physical observables and temporal/spatial averaging inherent to the two methods. Classical MD simulations describe atomistic configurations and fluctuations over femtosecond‐to‐nanosecond fluctuations and are subject to force‐field parameterization limitations, whereas Raman spectroscopy reflects a macroscopic ensemble‐averaged vibrational response. Thus, the two approaches are best interpreted as complementary, with their combined results supporting the conclusion that LiFSI promotes anion‐rich Li^+^ solvation structures relative to LiTFSI.

Gas‐phase DFT calculations show that FSI^−^ and TFSI^−^ anions possess similar intrinsic Li^+^‐coordinating strengths. Representative optimized structures of the solvent‐coordinated Li[DME]_3_ complex and the anion‐coordinated Li[anion][DME]_2_ complexes are depicted in Figure 3C. The calculated binding energies of Li[FSI][DME]_2_ and Li[TFSI][DME]_2_ are both approximately −720 kJ mol^−1^, substantially more favorable than that of the purely solvent‐coordinated Li[DME]_3_ complex, which is calculated to be −462 kJ mol^−1^ (Figure S7). These results suggest that, in the absence of bulk‐solvent effects, both anions are thermodynamically capable of replacing a coordinated DME molecule in the Li^+^ primary solvation sheath. However, this gas‐phase energetic similarity contrasts sharply with the experimental Raman results and MD‐derived solvation statistics, which reveal markedly different bulk solvation behaviors for LiFSI‐ and LiTFSI‐based electrolytes. This apparent discrepancy highlights that bulk solvation structures are not governed solely by isolated Li^+^–anion binding energies, but also by steric, packing, and solvent‐network effects. In particular, incorporation of the bulky relatively TFSI^−^ anion into the Li^+^ primary solvation shell likely incurs a substantial steric penalty. Its voluminous CF_3_– groups can generate unfavorable steric repulsion with neighboring DME molecules and disrupt the local solvent organization, thereby suppressing its participation in CIPs and AGGs despite its strong intrinsic Li^+^ affinity. By contrast, the smaller FSI^−^ anion can more readily access the Li^+^ coordination environment, enabling the anion‐rich solvation structures observed experimentally and computationally.

Given its exclusion from the primary solvation shell, the spatial distribution and local solvation environment of the NF additive warrant detailed examination. Both experimental and computational results confirm that NF contributes negligibly to the primary Li^+^ solvation sheath in LiTFSI‐ and LiFSI‐based electrolytes (Figures 2E and 3A). This is primarily attributed to its intrinsically weak electron‐donating ability. DFT calculations further reveal that artificially introducing NF into the Li^+^ coordination sphere to form Li[DME]_2_[NF] results in a less favorable binding energy than that of Li[DME]_3_ (−438 vs. −462 kJ mol^−1^). Furthermore, the interaction between nonpolar NF and strongly polar DME is substantially weaker than DME‐DME dipolar interactions, while NF‐NF self‐interaction is also minimal (Figures S8–S11, Supplementary Note 4#). Together, these features account for the limited intrinsic solubility of NF in neat DME solvent.

From an MD perspective, the substantial steric hindrance imposed by the nonafluorobutyl group restricts NF to a minimum separation of approximately 6 Å from Li^+^. The atomistic solvation environment of NF further corroborates this mechanism. As shown in the MD simulation snapshots for electrolytes containing 3 wt% NF (Figure 3D), NF is homogeneously dispersed throughout the bulk electrolyte. The average coordination environment within a 3.0 Å radius of NF over a 10 ns equilibrated MD trajectory was further quantified (Figures 3E and S12). In the LiTFSI‐based electrolyte, each NF molecule is surrounded, on an average, by 8.17 DME, 1.04 TFSI^−^, 0.13 NF, and zero Li^+^ species. In the LiFSI‐based electrolyte, the corresponding local environment comprises approximately 8.85 DME, 0.54 FSI^−^, 0.14 NF, and zero Li^+^ species. These results confirm that the trace NF additive is molecularly dispersed within the DME matrix, rather than forming local aggregates. This atomistic picture is further corroborated by ^19^F NMR longitudinal relaxation measurements (Figure S13). Specifically, the longitudinal relaxation time (T1) of neat NF is significantly shorter than those measured in the electrolyte systems, indicating that intermolecular coupling among NF molecules is effectively weakened upon dilution in both LiTFSI‐ and LiFSI‐based electrolytes.

By adopting NF as the central reference, a more comprehensive representation of the bulk solvation network can be established (Figure 3F). In both electrolytes, the center of the nearest Li^+^ solvation‐shell is located approxiamtely 6 Å from NF, while the closest neighboring NF molecule is separated by ca. 9 Å. This large intermolecular spacing underscores the highly dilute and well‐dispersed nature of NF within the electrolyte. Notably, TFSI^−^ anions exhibit a greater propensity to populate the local environment of NF compared to FSI^−^ anions. This distinction originates from their fundamentally differing solvation preferences in that while TFSI^−^ anions are largely expelled from the primary Li^+^ coordination shell and remain relatively mobile within the bulk DME network, FSI^−^ anions predominantly reside within the Li^+^ solvation structures, thereby limiting its availability to interact with dispersed NF molecules. These subtle yet consequential differences in cation‐anion solvation structures ultimately lead to different transport characteristics across the two electrolyte systems.

A statistical analysis of the anionic solvation environments, as depicted in Figure S14, further reinforces this interpretation. Although the average number of NF molecules in the immediate vicinity of any given anion remains relatively low, transient spatial encounters are consistently observed throughout the bulk solvent network. When these microscopic findings are correlated with the ^19^F NMR results discussed in Figure 2G, a coherent picture emerges, in that increasing the overall NF concentration systematically increases the average probability of NF molecules residing within the local anionic environment. This progressive modulation of the fluorine chemical environment directly accounts for the systematic upfield chemical shifts observed in the ^19^F NMR spectra. Collectively, these findings provide compelling atomistic evidence for the presence of weak, noncovalent interactions between NF and salt anions. Despite their modest strength, these ubiquitous NF‐anion interactions essentially act as an effective structural compatibilizer, thereby rationalizing the favorable macroscopic solubility and homogeneous miscibility of NF within the bulk electrolyte.

### 2.3 Statistical Treatment of Lithium Cycling Efficiency

To quantitatively investigate Li deposition/dissolution behavior occurring at the negative electrode compartments, asymmetric Li°||Cu° cells with NF‐containing electrolytes were subjected to galvanostatic cycling tests. Figure 4A compares the Aurbach CE values for the electrolytes with different amounts of NF additives. The details of the Aurbach protocol are discussed in Supplementary Note #1, and the source data of galvanostatic cycling profiles for multiple replicates are presented in Figure S15. For both LiTFSI‐ and LiFSI‐based electrolytes, the Aurbach CE values show minor changes in relation to the corresponding reference cells, e.g., 97.91% ± 0.27% (LF‐5N) vs. 97.13% ± 0.15% (LF‐0N) and 70.74% ± 0.93% (LTF‐5N) vs. 69.09% ± 1.66% (LTF‐0N), though the LiFSI‐based systems possess higher CE values attributed to the favorable SEI‐forming ability of the FSI^−^ anion. Clearly, the inclusion of NF additive in both electrolytes does not compromise the cycling stability of lithium electrodes in both electrolytes, instead, bringing somehow positive impacts for both systems. It has to be accentuated that the Aurbach CE values are indicative parameters for evaluating the compatibility of selected electrolytes with metal electrodes, specifically for short‐term cycling conditions (ca. spanning several days).

**FIGURE 4:**
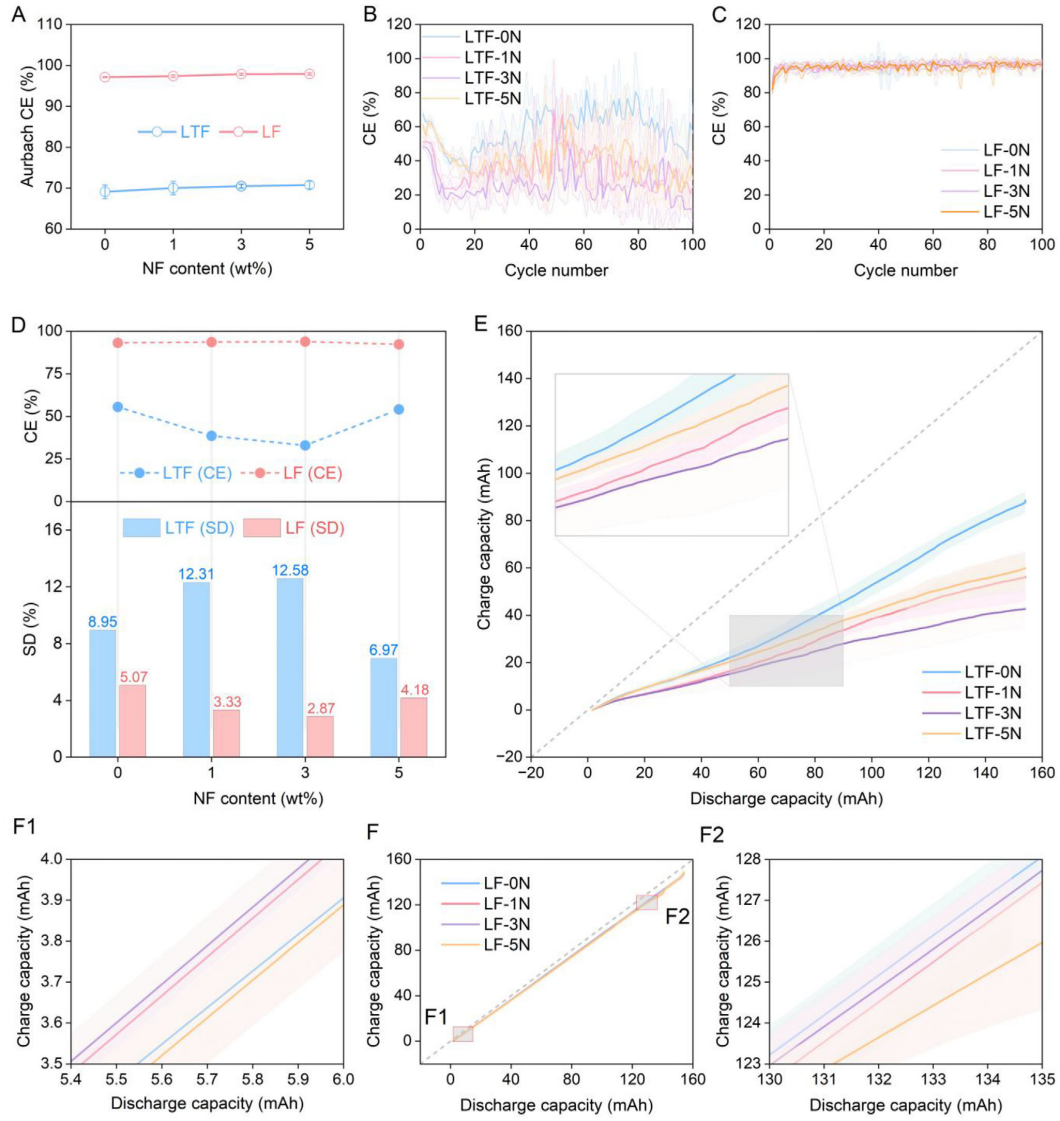
Lithium cycling efficiency in NF‐containing electrolytes. Quantitative evaluation of long‐term cycling‐derived Coulombic efficiency (CE) using statistical analyses. (A) Coulombic efficiency (CE) values determined by the Aurbach protocol for LiTFSI‐ and LiFSI‐based electrolytes. (B,C) Arithmetic mean of CE values with SD values (shaded region) over 100 cycles for the LiTFSI‐ (B) and LiFSI‐ (C) based electrolytes with various contents of NF additive. (D) Average CE and SD values calculated from the first ten cycles of the Li°||Cu° cells using different electrolytes. Current density: 0.5 mA cm^−2^, duration for charge/discharge processes: 2 hours. (E) Cumulative charge/discharge capacity for Li°||Cu° cells using LiTFSI‐based electrolytes with different amounts of NF additive. The inset figure shows the zoom‐in profile around cycle 50. **F**. Cumulative charge/discharge capacity for Li°||Cu° cells using LiFSI‐based electrolytes with different amounts of NF additive. The zoom‐in profiles around cycle 5 and 85 are provided in the left (F1) and right (F2) panels, respectively. In Figures 4B, 4C, 4E and 4F, solid lines represent the arithmetic mean values, while the shaded regions correspond to the standard deviation (SD) obtained from four parallel cells.

To reveal the long‐term impact brought by the NF additive, asymmetric Li°||Cu° cells were subjected to long‐term cycling, and the as‐obtained cycle‐by‐cycle CE values were statistically analyzed. Figure 4B,C present the evolution of CE values over 100 cycles for the two electrolyte systems. In these plots, the dotted line denotes the arithmetic mean of CE values, while the shaded region indicates the variation range defined by the standard deviation (SD). In the absence of NF additive, the Li°||Cu° cells show significantly better cycling stability by replacing the TFSI^−^ with FSI^−^ anion, and the later families show CE values higher than 80% for each cycle (Figure 4C). With extended cycles, the two electrolyte systems show more pronounced differences in CE and SD values, allowing more precise quantification of lithium cycling efficiency and the long‐term influence of NF incorporation.

Focusing on the initial cycling behavior, Figure 4D displays the average CE values calculated from the first 10 cycles, together with the corresponding SD values. For the LiTFSI‐based electrolyte, the addition of NF additive generally results in slight decrease in average CE values, as compared to the reference cell [e.g., 32.96% (LTF‐3N) vs. 55.56% (LTF‐0N)]. Meanwhile, the SD value increases slightly from 8.95% to 12.58% upon the addition of 3 wt% NF. These results indicates that the presence of NF additive is unfavorable for achieving stable lithium cycling in LiTFSI‐based electrolytes. This is further evidenced by the cumulative charge/discharge capacity plot shown in Figure 4E. It is important to note that the cumulative capacity values are obtained by summing (dis) charge capacities accumulated over each cycle, and the diagonal line indicates 100% reversibility of each cycle, as detailed in our previous work [23]. For the NF‐added cell, the cumulative capacity profile deviates largely from the diagonal line after nearly 50 cycles, due to the ever‐decreasing cumulative charge capacity. Eventually, an exceedingly large amount of discharge capacity cannot be recovered during the charge process, corresponding to a cumulative capacity loss of 111.2 mAh for LTF‐3N vs. 65.5 mAh for LTF‐0N after 100 cycles (Figure 4E). These results evidence the negative impact brought by NF additive for long‐term cycling with LiTFSI‐based electrolytes, which cannot be directly deduced from the Aurbach CE tests.

Returning to the initial cycling behavior for LiFSI‐based electrolytes (Figure 4C), the incorporation of an optimized NF additive (i.e., 3 wt%) increases the average CE values from 93.20 to 93.90% while simultaneously reducing the SD values. This testifies the positive influence of coupling NF additive with FSI^−^ anions for lithium cycling, as also confirmed by the cumulative capacity profiles for the first few cycles, in which a smaller deviation from the diagonal line is noticed after adding NF additive (Figure 4F). However, upon extending the cycling duration, the positive impact of NF additive diminishes, and the deviations of cumulative capacity profiles become comparable for the samples with and without NF additive [ca. 7.2 mAh (LF‐3N) vs. 6.7 mAh (LF‐0N) for the cumulative capacity loss after 100 cycles, Figure 4F].

These observations suggest that the incorporation of NF additive has only a marginal impact on the Li deposition/dissolution processes occurring on the Cu° substrate. Consequently, it makes it difficult to clearly discern the effect of the NF additive on SEI formation using conventional CE analysis of asymmetric Li°||Cu° cells alone. Fortunately, the application of statistical analysis to long‐term cycling data provides more explicit insights into the CE behavior. It is likely that the reductive decomposition of FSI^−^ anions is more pronounced than that of TFSI^−^ anions, and their interaction with the NF additive is highly dependent on the electrochemical conditions. This highlights the trade‐off between NF‐promoted SEI formation and excessive electrolyte decomposition during SEI growth and Li° deposition.

To further evaluate the practical relevance of the electrolyte design, the Cu°||LiFePO_4_ cells were assembled and tested (Figure S16). In consistent with the Li°||Cu° results, the LiFSI‐based electrolytes exhibit substantially improved electrochemical performance in the Cu°||LiFePO_4_ cells, as compared to LiTFSI‐based electrolytes, which is attributed to the stronger SEI‐forming ability of the FSI^−^ anions [42]. For the LiTFSI‐based electrolyte, the addition of NF additive slightly increases the charge capacity for the second cycle (Figure S16h), suggesting that the NF additive can partially protect deposited Li° from continuous side reactions with electrolyte components (solvents and salt anions). Yet, the scenario changes drastically in the LiFSI‐based electrolytes, and the addition of NF additive consumes active lithium ions from the LiFePO_4_ cathode, slightly reducing the charge capacity of the second cycle (Figure S16h). This is possibly due to the competitive reductions of NF and FSI^−^ anions on the surface of Cu° electrode after Li° deposition. These experimental evidences further reveal the interplay between lithium protection and SEI formation/reconstruction for sustaining reversible cycling of Li° negative electrodes in a working cell [43].

### 2.4 Li Deposition Behavior and Interphasial Compositions

To evaluate the morphological changes induced by the NF additive on the working Li° electrode, scanning electron microscope (SEM) experiments were performed on Li° deposits recovered from LiTFSI‐ and LiFSI‐based electrolytes. Figure 5A compares the surface morphologies of Li° deposits obtained with different electrolyte formulations. In the absence of NF additive, the surface of Li° deposits exhibits a loose and porous structure for LiTFSI‐based electrolyte, featuring mossy and dendritic lithium with localized sharp protrusions (Figure 5A_1_). By replacing LiTFSI with LiFSI, the Li° deposits become more uniformly distributed and the cracks and porous structure on the Li° surface are effectively reduced (Figure 5A_3_). This clearly suggests the effectiveness of the FSI^−^ anion in regulating the surface morphology of Li deposits, as extensively discussed in previous work [42]. The surface patterns obviously change after adding a small portion of NF additive (3 wt%). As can be seen in Figure 5A_2_ and 5A_4_, the Li° deposits recovered from both LiTFSI‐ and LiFSI‐based electrolytes become more compact and smoother in the presence of NF additive. In particular, in the LiFSI‐based sample, the presence of NF promotes the growth of larger crystalline particles [ca. 10 µm (LF‐3N) vs. 3 µm (LF‐0N), Figure 5A_4_]. These above‐morphological changes indicate that the salt anion and NF additive acts synergistically to regulate the nucleation and growth of Li deposits and thus their structure and morphology.

**FIGURE 5:**
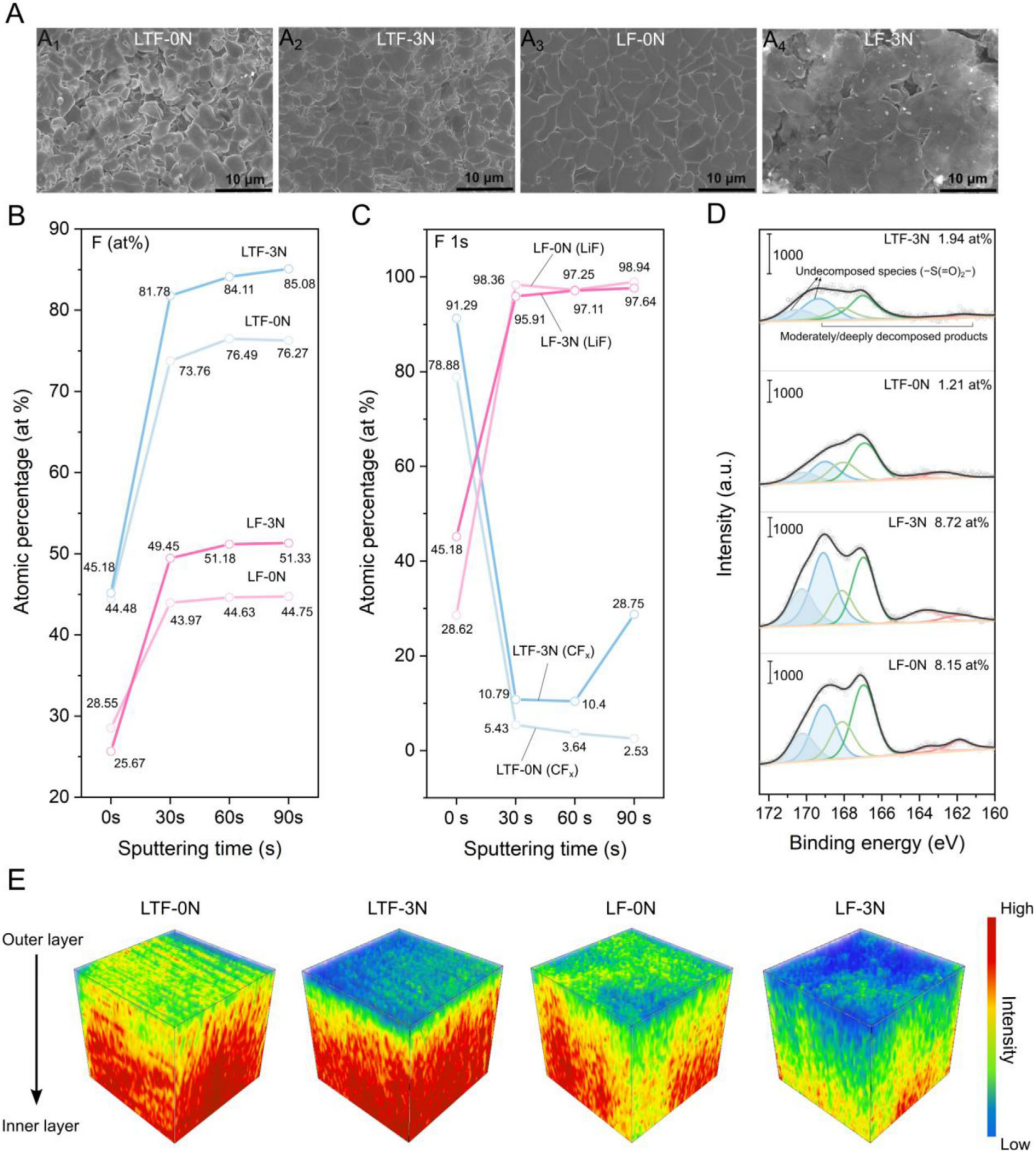
Lithium deposition morphology and interphasial compositions. (A) SEM images of Li° deposits in LiTFSI‐based (A_1_, A_2_) and LiFSI‐based (A_3_, A_4_) electrolytes. (B) Atomic percentages of the F‐containing species under different sputtering time derived from the corresponding XPS survey spectra. (C) Dependence of the atomic percentage of CF*_x_*‐containing species in LiTFSI‐based electrolytes and LiF in LiFSI‐based electrolytes with sputtering time, determined from the deconvoluted high‐resolution F 1s spectra. (D) High‐resolution S 2p spectra recovered from the LiTFSI‐ and LiFSI‐based electrolytes at the end of sputtering time (i.e., 90 seconds). The atomic percentages of total sulfur‐containing species shown in the figure are obtained from the XPS survey spectra (Figure S17). (E) Reconstructed 3D TOF‐SIMS images showing the spatial distribution of LiF^−^ signals in Li° deposits recovered from the LiTFSI‐ and LiFSI‐based electrolytes (sputtering time 150 seconds).

To characterize the chemical compositions of the Li° deposits, X‐ray photoelectron spectroscopy (XPS) measurements were further performed on samples prepared using both LiTFSI‐based and LiFSI‐based electrolytes with an optim amount of NF additive (3 wt%). The areal capacity for lithium deposition was fixed at 2 mAh cm^−2^, and the XPS survey spectra are presented in Figure S17. A list of characteristic peaks originating from the constituent elements of the lithium salt, solvent, and NF additive is observed (Figures S18, S19), and their assignments are provided in Table S3. For example, species containing C─O bonds (ca. 286.4 eV) observed in the C 1s spectra (Figure S18) are majorly sourced from the ether solvent, whereas the fluorinated species are generally inherited from salt anion and NF additives [e.g., CF_x_ (688.5 eV), LiF (684.8 eV) in the F 1s spectra, Figure S19].

Figure 5B presents atomic percentages of fluorine (F) on lithium deposited from four different electrolytes under different sputtering times. For the four samples, the atomic percentage of F remains lower than 50 at% and increases significantly after a short sputtering treatment (i.e., 30 s). The values are stabilized when extending the sputtering time from 60 to 90 seconds, which indicates the gradual exfoliation of the porous organic outer layer, accompanied by exposure of the inner SEI layer primarily composed of inorganic species generated via anion decomposition and NF fragmentation. In the reference electrolytes (i.e., without NF additive), due to the intrinsic difference in F content between TFSI^−^ and FSI^−^ anions, LiTFSI‐based electrolyte exhibits a higher atomic proportion of F‐containing species as compared to LiFSI‐based one (Figure 5B). After introducing NF additive, F‐containing species increase slightly for both electrolyte systems, e.g., 76.27 at% (LTF‐0N) vs. 85.08 at% (LTF‐3N) and 44.75 at% (LF‐0N) vs. 51.33 at% (LF‐3N) at the end of sputtering treatment (i.e., 90 seconds). This suggests that the decomposition and reduction patterns of electrolyte components are partially regulated in the presence of NF additive, thereby effectively tuning the chemical compositions of the as‐formed SEI layer. That is, such processes are partially overlapping with the reductive decompositions of salt anions, highlighting the potential synergy between electrolyte additives and electrolyte salts.

Figure 5C shows the atomic percentages of F‐related species deconvoluted from the corresponding high‐resolution F 1s spectra. LiF appears as a common inorganic decomposition product in both LiTFSI and LiFSI‐based samples due to the reductive fragmentation of these two fluorinated anions. The organic CF*_x_*‐containing species stem from the incomplete decompositions of TFSI^−^ anion, usually inherited from the pristine trifluoromethanesulfonyl group (CF_3_SO_2_─). These CF*_x_*‐containing species may originate from partially reduced TFSI‐derived fragments, such as trifluoromethanesulfinyl group (CF_3_SO─) with a lower valence state sulfur atom, and partially defluorinated difluoro‐/monofluoro moieties (e.g., ─SOCF_2_─ or ─SOCF─), depending on the degree of reductive decompositions [44]. For the LiTFSI‐based electrolyte, the outer SEI layer exhibits a much higher atomic proportion of CF_x_‐containing species than the inner SEI layer. The introduction of NF additive in LiTFSI‐based electrolyte seems to enrich the formation of organic fluorinated components and causes a relatively porous structure in the inner SEI layer [45]. For example, after a slightly extended sputtering of 90 seconds, the CF_x_‐containing species ratio increases from 2.53 at% (LTF‐0N) to 28.75 at% (LTF‐3N), as also evidenced by the high‐resolution C 1s spectra (cf. Figure S20). For LiFSI‐based electrolytes, in the absence of NF additive, the inner SEI layer exhibits a remarkably higher LiF ratio as compared to the outer layer, suggesting the as‐formed inner SEI layer is dominated by anion decomposition, and the decomposition products derived from FSI^−^ are dominated by inorganic species. After adding the NF additive, the atomic percentage of LiF in the inner SEI layer reduces, accompanied by an increased proportion in the outer SEI layer [e.g., 28.62 at% (LF‐0N) to 45.18 at% (LF‐3N) without sputtering; 98.94 at% (LF‐0N) to 97.64 at% (LF‐3N) after 90 s sputtering]. These results demonstrate that NF additive regulates the spatial distribution of LiF in the as‐formed SEI layer, which effectively balances the mechanical and transport properties throughout the inner and outer SEI layer.

Figure 5D shows the high‐resolution S 2p spectra and corresponding atomic percentage of S‐containing species after 90 s sputtering obtained from the four electrolytes. With respect to the S 2P spectra, sulfur‐containing species majorly originating from incomplete decompositions of both TFSI^−^ and FSI^−^ anions and NF additive are clearly observed, enlisting sulfonyl moieties (e.g., –S(=O)_2_–, sulfur (VI), 169.0–170.5 eV), sulfinate groups ((e.g., –S(=O)–, 167.0–168.1 eV) with medium‐valence state sulfur (IV) and fully reduced states as elemental and sulfide species (e.g., S^0^/S_x_^2−^). It is noteworthy to note that NF additive also contains fluorosulfonyl (FSO_2_–) and fluoroalkyl (R_f_–) moieties, which can generate sulfur‐related species with different valence states and LiF during its participation in SEI formation (cf. Figure 6).

**FIGURE 6:**
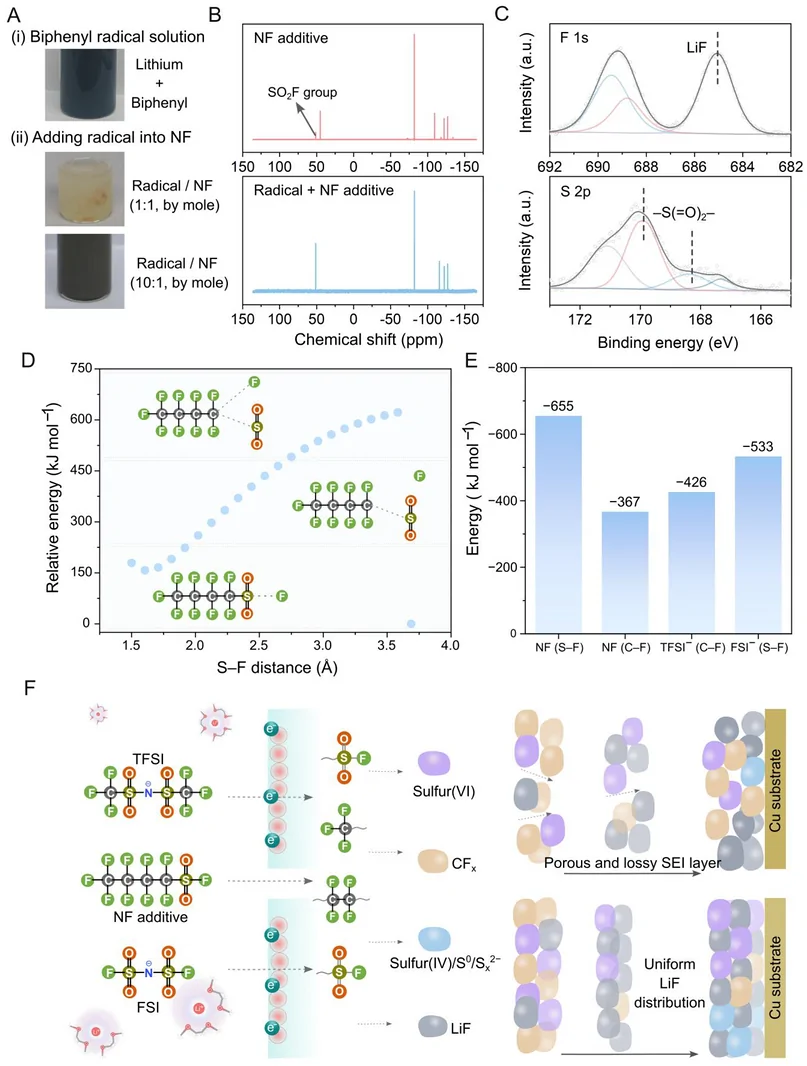
Chemical simulation and theoretical calculation of the decomposition process of NF additives. (A) Evolution of physical appearance of biphenyl radical solutions with different contents of NF additive. (B) ^19^F NMR spectra of the soluble phase. (C) High‐resolution F 1s and S 2p XPS spectra of the solid phase. (D), (E) DFT calculations of decomposition mechanism of the NF additive at the atomic scale. Reaction energies corresponding to S─F bond cleavage in NF additives (D), and C─F/S─F bond dissociation in TFSI^−^ anions, FSI^−^ anions, and NF additives, respectively (E). (F) Schematic diagram of decomposition mechanism for LiTFSI‐ and LiFSI‐based electrolytes and NF additives.

Without the addition of NF additive, the sulfur‐containing species tend to be higher for the LiFSI‐based sample in the inner SEI layer, possibly SEI building compounds such as Li_2_S, Li_2_SO_3_, and Li_2_SO_4_ compounds, as compared to the LiTFSI‐based one [e.g., 1.21 at% (LTF‐0N) vs. 8.15 at% (LF‐0N)], which suggests that the FSI^−^ anion can be actively decomposed during the inner SEI layer formation. With the addition of NF additive, the atomic percentage of sulfur‐containing species slightly increases for both LiTFSI‐ and LiFSI‐based electrolytes after 90 s sputtering, suggesting the participation of NF additive in SEI‐formation processes at the very early stages. From the deconvoluted S 2p spectra (Figure 5D), the enrichment of sulfur‐containing species in high‐valence states (i.e., S(VI)) tends to be less pronounced in the LiFSI‐based sample after the incorporation of NF additive [e.g., 31.53 at% (LTF‐0N) vs. 45.47 at% (LTF‐3N) for LiTFSI system, whereas 40.56 at% (LF‐0N) vs. 50.11 at% (LF‐3N) for LiFSI system], indicating that FSI^−^ anion compete more actively with NF additive in the inner SEI formation. Furthermore, the incomplete decomposition of NF additive leads to a higher retention of S(VI) and organic CF_x_ species in the inner SEI layer, which hinders further decomposition of FSI^−^ anions. The above results indicate that the NF additive can partially mitigate the severe decomposition of FSI^−^ anions on fresh lithium metal and regulate the spatial distributions of the decomposition products from the FSI^−^ anions. Combining with the atomic percentage of F‐containing species, the introduction of NF enables a reinforcing balance between organic and inorganic components in the inner SEI layer of the LiFSI‐based electrolyte, which contributes to the improved deposition stability of Li°||Cu° cells in the early cycling stage.

Figure 5E shows the reconstructed 3D images based on the time‐of‐flight secondary ion mass spectrometry (ToF‐SIMS) experiments, illustrating the spatial distribution of LiF component measured with four different electrolytes. Connecting to the XPS results, a sharp increase in LiF intensity is observed for all measured samples by extending the sputtering time, which is primarily due to the reductive decomposition of fluorinated NF additive and salt anions at early SEI‐forming stages. After adding NF additive, the spatial distribution of LiF in the outer SEI layer becomes more uniform for both LiTFSI‐ and LiFSI‐based electrolytes, while local enrichment of LiF is suppressed specifically for the latter salt‐based electrolyte. This further testifies that the NF additive is involved in the SEI‐forming processes and its synergy with salt anions is critical for the ion transport and electrochemical reactions at the negative electrode compartment (cf. Figure 4).

### 2.5 Chemical Mechanism of NF Additive

To gain mechanistic insights into the interfacial behavior of the NF additive and its synergy with conducting salts, biphenyl radical anion (Ph_2_•^−^) was employed to chemically simulate their reductive decomposition on a lithium metal electrode. Such chemical mimicry of susceptibility to electrochemical reduction was successfully applied to lithium bis(trifluoromethanesulfonyl)imide, lithium (fluorosulfonyl)(trifluoromethanesulfonyl)imide, and lithium bis(fluorosulfonyl)imide salts in our previous work [19]. The experimental workflow used for these chemical simulations is shown in the Experimental details. After the radical reactions, species soluble in ethereal solvents were analyzed by ^1^H and ^19^F NMR, while the insoluble products were characterized by XPS to identify their chemical compositions. Figure 6A shows the evolution of the biphenyl radical solutions’ appearance upon addition of varying amounts of NF additive. The pristine biphenyl mono‐radical solution exhibits the characteristic intense blue color, which immediately turns colorless upon the addition of an equimolar amount of NF additive. The rapid color change indicates the cleavage of polar bonds within the NF additive by the highly reducing biphenyl radicals. When excess biphenyl radical is added, the solution reverts to deep blue, demonstrating that nearly all reactive bonds in the NF additive have been consumed.

Figure 6B presents the ^19^F NMR spectra of the soluble phase recovered from the reaction between excess biphenyl radical and NF additive, alongside the reference spectrum of pristine NF additive. In the reference spectrum, the FSO_2_– group appears at a chemical shift of +50 ppm; however, this signal is absent after the radical reaction (Figure 6B). This disappearance indicates that the decomposition products originating from the FSO_2_– moiety of NF precipitate from the ethereal solvent, likely due to their inorganic nature (i.e., LiF, LiSO_2_F) [46]. In addition, minor peaks between −122 and 130 ppm can be attributed to species containing CF_x_ moieties, likely originating from the pristine or partially decomposed nonafluorobutyl (C_4_F_9_─) groups in the NF additive. Figure 6C presents high‐resolution F 1s and S 2p XPS spectra of the insoluble products recovered from chemical simulation, while the full survey spectra are provided in Figure S21. In the F 1s spectra, the peak at 685.0 eV is characteristic of LiF, while those at higher binding energies correspond to CF*_x_* (688.8–689.5 eV) species. The LiF in the insoluble phase results from the precipitation of F^−^ ions released during the chemical cleavage of the S─F bond in NF additive. This observation corroborates the ^19^F NMR spectra of the supernatant liquid phase (Figure 6B), where F^−^ signal in the −150 ppm to −180 ppm region is barely detectable. In the S 2p spectra, two peaks appear at 169.9 eV and 168.3 eV, corresponding to the ─S(═O)_2_─ group and ─S(═O)─O─ groups, respectively. The coexistence of S(VI) and S(IV) in the S 2p spectra clearly indicates that the decomposition of NF additive is relatively incomplete, in contrast to classic SEI‐forming salt additives such as FSI^−^ anions. This is likely due to the slower decomposition kinetics of the FSO_2_─ groups in the molecular compound (i.e., NF) compared with the ionic ones (i.e., FSI^−^). The incomplete reductive decomposition of NF additive under an excess amount of biphenyl radical is further supported by the C 1s spectra, where the two peaks corresponding to CF*_x_*‐containing species are observed at 290.5 eV and 292.1 eV (Figure S22).

To elucidate the decomposition mechanism of the NF additive at the atomic scale, DFT calculations were performed with particular attention to the bonding characteristics of carbon‐fluorine (C─F) and sulfur‐fluorine (S─F) linkages. Figure 6D shows the energy evolution of the NF additive upon deliberate elongation of the S─F bond, with all other atoms relaxed. At a bond length of 1.6 Å, NF resides in its lowest energy state, with a relative energy of 157 kJ mol^−1^. As the S─F bond is gradually stretched from 1.6 Å to ca. 3.6 Å, the relative energy increases continuously, reaching a maximum of ca. 622 kJ mol^−1^, reflecting the energetic barrier associated with the bond stretching and initial structural distortion of the parent molecule. Once the S─F bond exceeds 3.6 Å, the relative energy drops sharply from 622 kJ mol^−1^ to 0 kJ mol^−1^, indicating a thermodynamically favorable transition to the decomposed state, where the resulting decomposition products are more stable than the original NF molecule. Concomitant with these energy changes, distinct structural transformations occur, including extrusion of SO_2_, and the formation of new carbon‐fluorine (C─F) bonds that generate nonafluorobutane (C_4_F_10_). These observations are consistent with previous reports on the chemical reactivity of NF in the presence of organic bases, in which NF readily decomposes into C_4_F_10_ and SO_2_ [47].

For the generation of LiF via the decomposition of electrolyte additives or salt anions, the key process lies in the formation of fluoride ions. Figure 6E further compares the energy changes associated with the abstraction of one fluorine atom from the NF additive, TFSI^−^ and FSI^−^ anions in the presence of two lithium atoms. As shown in Figure 6E, fluorine abstraction from the S─F bond exhibits more favorable thermodynamic changes compared to that from the C─F bond, e.g., −533 kJ mol^−1^ (FSI^−^ anion) vs. −426 kJ mol^−1^ (TFSI^−^ anion). For the NF additive, the same dissociation pattern is observed, where S─F bond cleavage affords an additional stabilization energy exceeding 250 kJ mol^−1^, i.e., −655 kJ mol^−1^ (S─F) vs. −367 kJ mol^−1^ (C─F). This is consistent with the lability of S–F linkages testified by the electrochemical tests. It has to be accentuated that the S─F bond in the NF additive tends to be more readily abstracted by lithium atoms relative to that in the FSI^−^ anion, indicating higher chemical reactivity of NF under electrochemical conditions. In contrast to the homolytic or heterolytic dissociation of the neat compounds, negative energy values are consistently obtained in the presence of atomic lithium. This is a clear indication of dual decomposition pathways for the NF additive on the surface of lithium metal electrodes.

Based on the above experimental and computational results, the working mechanism of NF additive toward SEI formation at the Li° electrode is schematically illustrated in Figure 6F. For the prevalent TFSI^−^ anions, the two equivalent trifluoromethyl moieties covalently bonded to the high‐valence Sulphur are highly resistant to releasing fluorine atoms due to the high thermodynamic stability of the C─F bonds in fluorinated alkyl chains. During Li deposition and dissolution processes, TFSI^−^ anions are less readily reduced at the Li° surface in relation to FSI^−^ anions, leaving active sites available for ethereal solvents. Consequently, a porous and organic‐rich SEI layer forms, as extensively reported in previous studies [42]. This implies that, in the LiFSI‐based electrolyte, solvent decomposition leading predominantly to organic SEI species is suppressed due to effective passivation by LiF formed at higher potentials. In contrast, in LiTFSI‐based electrolytes, the stronger C─F bonds hinder LiF formation, resulting in incomplete passivation and consequently continuous solvent reduction.

FSI^−^ anion is considered a “magic” SEI precursor, and its unique role in SEI‐building processes has attracted significant attention in the battery community, as highlighted in several recent studies [48, 49, 50]. The two S─F bonds are highly reactive and prone to electrochemical reduction, serving as fluoride precursors for the construction of a LiF‐rich SEI layer. Cleavage of these S─F bonds induces structural decomposition of the FSI^−^ anions, concurrently generating sulfur‐containing species with multiple valence states (i.e., sulfate, sulfinate, sulfide, etc.). These inorganic species are critical for maintaining the mechanical integrity of the SEI layer on Li° electrodes, while limiting electron leakage. Consequently, replacing conventional TFSI^−^ with FSI^−^ anion in liquid and/or solid electrolytes leads to higher CEs and longer cycle life [10].

With respect to the NF additive, the characteristic FSO_2_– functionalities from the FSI^−^ anion are simultaneously integrated into the same molecule alongside the typical CF_3_– groups in TFSI^−^ anions. In the FSO_2_– moiety, bonding to a positively charged carbon, rather than the negatively charged nitrogen in FSI^−^ anion, kinetically facilitates more facile electrochemical decomposition. However, this decomposition pathway competes with that of FSI^−^ and is strongly influenced by the precedent surface species covering the lithium deposits. Once the FSO_2_– and C_4_F_9_– functionalities in NF additive are decomposed, the lithium surface will be passivated, and further decomposition of FSI^−^ anions will be practically suppressed. This is effectively beneficial for mitigating the “explosive” decompositions of the FSI^−^ anion without NF additives, which aligns with the slightly improved CE values in the corresponding Li°||Cu° tests. Since NF additives are gradually consumed during reconstruction of SEI, its impact becomes diminished with extensive cycles (cf. Figure 4F).

Conversely, organic fluorinated organic species are readily generated from the decomposition of either NF or TFSI^−^ anions, since both components contain relatively high content of fluoroalkyl groups. The dissolution and re‐precipitation of organic species cause the formation of porous and mossy SEI layers, which can barely prevent electrolyte penetration and further degradation. This manifests as significant fluctuations in the CE values after NF incorporation in the LiTFSI‐based samples (cf. Figure 4B).

Overall, the addition of NF into the LiTFSI‐ and LiFSI‐based electrolytes brings significantly diverging impacts in terms of lithium cycling efficiency. The competitive decomposition of NF additive and FSI^−^ salt anion contributes to better spatial distribution of LiF over the entire SEI layer, thus balancing the mechanical and transport properties and facilitating higher reversibility for lithium cycling at initial stages. While the excessive amount of organic fluorinated species stemming from NF additive is detrimental to reversible lithium plating/stripping in the LiTFSI‐based system, it adversely impacts the cycling stability of lithium electrode. More broadly, translating the tantalizing features of specific additives into desirable SEI properties requires synergistic optimizations of the electrolyte solvent and salt anions.

## 3 Conclusion

In summary, this work elucidates the nuanced role of the fluorinated additive nonafluorobutanesulfonyl fluoride (NF) in regulating the cycling stability of Li° electrodes by integrating statistical analysis of long‐term Li°||Cu° cycling data, advanced characterization, and computational simulations. We show that, at moderate loadings below 7 wt%, NF is homogeneously distributed in ether‐based electrolytes containing sulfonimide anions and induces only a limited perturbation of the primary Li^+^ solvation structure. Although FSI^−^ anions are highly involved in the cation solvation sheath, a small portion of NF additive (<1 wt%) leads to a lower degree of microscopic discontinuity and minimizes the decrease in ionic conductivity. In addition, statistical analysis of CE values obtained from long‐term Li°||Cu° cycling reveals an electrolyte‐dependent effect of NF that is not captured by conventional Aurbach CE measurements: NF improves cycling reversibility in the LiFSI system while deteroriating performance the LiTFSI system. This divergence arises from competitive interfacial decomposition among NF and fluorinated sulfonimide anions, which governs the spatial distribution and chemical uniformity of fluorinated SEI components. In particular, incorporating NF into FSI‐based electrolytes promotes a more even distribution of fluorine‐rich decomposition products on the Li° electrode, thereby stabilizing the interphase and improving cycling performance.

More broadly, our findings caution against assigning additive functionality solely on the basis of intuitive chemical motifs, such as the fluorosulfonyl group in NF. Instead, effective electrolyte design requires a holistic consideration of additive‐anion‐solvent interactions, coupled with statistically robust electrochemical protocols capable of resolving the spatiotemporal evolution of interphase formation. That is to say, CE should not be interpreted as the simple consequence of a single electrolyte component or additive. Rather, it arises from the complex interplay among all electrolyte species, including additives, anions, and solvents, as well as their coupled reinforcing and/or counteracting interfacial decomposition pathways, which collectively govern SEI formation, evolution, and the structure–property–function relationships of the interphase. These findings refine the mechanistic understanding of electrolyte additives, bridge the gap between molecular structure and interfacial function, and provide design principles that can be applicable to other alkali‐metal battery chemistries, including sodium and potassium batteries.

## Author Contributions

**Qixin Wang**: writing – original draft, investigation, validation, data curation, writing – review and editing. **Jiaxun Yang**: writing – original draft, writing – review and editing, data curation. **Pu Li**: writing – original draft, data curation, writing – review and editing. **Kun Zhao**: investigation, writing – review and editing. **Xinxin Zhang**: investigation, writing – review and editing. **Bowen Zheng**: investigation, writing – review and editing. **Qiangfu Sun**: investigation, writing – review and editing. **Zhongyang Zhang**: investigation, writing – review and editing. **Wenfang Feng**: writing – review and editing, supervision. **Javier Carrasco**: writing – review and editing, supervision, funding acquisition. **Hailong Yu**: writing – review and editing, supervision, funding acquisition. **Gebrekidan Gebresilassie Eshetu**: writing – review and editing, funding acquisition, supervision, project administration, writing – original draft. **Egbert Figgemeier**: writing – review and editing, supervision. **Michel Armand**: writing – review and editing, supervision. **Xuejie Huang**: supervision, writing – review and editing. **Zhibin Zhou**: writing – review and editing, supervision. **Heng Zhang**: writing – review and editing, project administration, resources, writing – original draft, supervision, funding acquisition.

## Conflicts of Interest

The authors declare no conflicts of interest.

## Supporting information




**Supporting File**: anie73840‐sup‐0001‐SuppMat.pdf.

## Data Availability

The data that support the findings of this study are available from the corresponding author upon reasonable request.
