# Supplementary material for: Revisiting Coulombic Efficiency Paradigm: Electrolyte Additive Design for Lithium Metal Batteries
Source: Angew Chem Int Ed Engl. 2026 Jul 31;65(40):e9595705. doi: 10.1002/anie.9595705 (PMC13618272; doi:10.1002/anie.9595705)
Supplement: Supplementary file 1 [file ANIE-65-e9595705-s001.pdf]

Supporting Information

Revisiting Coulombic Efficiency Paradigm: Electrolyte Additive Design for Lithium Metal Batteries

Qixin Wang,[1,#] Jiaxun Yang,[1,#] Pu Li,[1,#] Kun Zhao,[1] Xinxin Zhang,[2] Bowen Zheng,[2] Qiangfu Sun,[2] Zhongyang Zhang,[2] Wenfang Feng,[1] Javier Carrasco,[3,4] Hailong Yu,[2,*] Gebrekidan Gebresilassie Eshetu,[5,6,*] Egbert Figgemeier,[5,7,8] Michel Armand,[3] Xuejie Huang,[2] Zhibin Zhou,[1] and Heng Zhang[1,*]

[1]Key Laboratory of Material Chemistry for Energy Conversion and Storage (Ministry of Education), School of Chemistry and Chemical Engineering, Huazhong University of Science and Technology, Wuhan, China.

[2]Institute of Physics, Chinese Academy of Sciences, Beijing, China.

[3]Centre for Cooperative Research on Alternative Energies (CIC energiGUNE), Basque Research and Technology Alliance (BRTA), Vitoria-Gasteiz, Spain.

[4]IKERBASQUE, Ikerbasque, Basque Foundation for Science, Bilbao, Spain.

[5]Institute of Power Electronics and Electric Drives (ISEA), Center for Ageing Reliability and Lifetime Prediction of Electrochemical and Power Electronic Systems (CARL), RWTH Aachen University, Aachen, Germany.

[6]Department of Material Science and Engineering, Mekelle Institute of Technology, Mekelle University, Mekelle, Ethiopia.

[7]Helmholtz Institute Münster (HI MS), Institute of Energy Materials and Devices (IMD-4), Forschungszentrum Jülich GmbH, Münster, 48149, Germany

[8]Jülich Aachen Research Alliance, JARA-Energy, Jülich, Germany.

[#] Q.W., J.Y., and P.L. contributed equally to this work.

* E-mail: yuhailong@iphy.ac.cn, Gebrekidan.Eshetu@isea.rwth-aachen.de; hengzhang2020@hust.edu.cn

WILEY-VCH

1. Supplementary Methods

1.1 Materials

Lithium salts including LiTFSI (battery grade, Solvay) and LiFSI (battery grade, Wuhan Revo-Energy, China), were used after drying under vacuum for 12 hours at 80 °C. The battery grade materials including NF (nonafluorobutanesulfonyl fluoride, Sinopharm Chemical Reagent, China) and Li° foil (99.95%, China Energy Lithium, 500 μm in thickness and 14.0 mm in diameter) were used as received. The $LiFePO_4$ cathode laminate was made using 95.0 wt% $LiFePO_4$ (Shenzhen Kejing, China), 2.5 wt% polymeric binder [poly(vinylidene fluoride), Arkema], and 2.5 wt% conductive carbon (carbon nanotube, Suzhou First Element Nanotechnology Co., LTD, China). The $LiFePO_4$ laminate (ca. 13 mg cm^{-2} of active material, ca. 110 μm in thickness excluding aluminum current collector) was punched into disks with a diameter of 14.0 mm and dried under vacuum for 12 hours at 100 °C before used. The polypropylene membrane (Celgard 2400, Shenzhen Kejing, China, 16.2 mm in diameter) and copper foil (Shenzhen Kejing, China, 15.0 mm in diameter) were first rinsed with ethanol and deionized water to remove surface impurities, then dried under vacuum at 60 °C for 8 hours. After this drying process, both materials were used directly without further treatment. 1,2-Dimethoxyethane (DME, analytical reagent, Sinopharm Chemical Reagent, China), diethylene glycol dimethyl ether (diglyme, analytical reagent, Sinopharm Chemical Reagent, China) and tetrahydrofuran (THF, analytical reagent, Sinopharm Chemical Reagent, China) solvents were purified according to standard procedures [1].

1.2 Electrolyte Preparation

The LiTFSI- and LiFSI-based electrolytes (salt concentration: 1.0 mol L^{-1}) were prepared by dissolving the required amount of lithium salt in the solvent (DME) using a volumetric flask in argon-filled glove box (H_2O, O_2 < 0.1 ppm, Mikrouna, China). Electrolyte formulations with different amounts of NF content (denoted as LTF-*x*N, x = 0, 1, 3, and 5 wt%) were prepared by adding the desired mass to the parent electrolytes. The water content in the resulting electrolytes was quantified by Karl–Fischer titration (Metrohm KF831), and the values were lower than 30 ppm.

1.3 Characterization

Ionic conductivities of the as-prepared electrolyte systems were measured by electrochemical impedance spectroscopy (EIS) using a multi-channel Metrohm electrochemical workstation (Multi Autolab/M204). Measurements were conducted over a frequency range of 10 to 10^6 Hz with an AC signal amplitude of 5 mV. All samples were tested three times independently, and the final ionic conductivity values are reported as the mean ± standard deviation (SD).

The phase transitions of the obtained electrolytes were characterized using differential scanning calorimetry (DSC) with measurements acquired on a NETZSCH DSC200F3 instrument.

WILEY-VCH

Raman spectra of the neat solvent as well as lithium-based electrolytes were collected using a laser confocal Raman spectrometer (LabRAM HR800, Horiba Jobin Yvon). The samples were sealed in glass capillaries, and a 532 nm excitation laser was employed for all measurements.

The chemical shift changes in LiTFSI- and LiFSI-based electrolytes were analyzed by nuclear magnetic resonance (NMR) spectroscopy using a Bruker AV400 spectrometer. Coaxial NMR tubes were employed to prevent direct contact between the samples and the deuterium reagent (i.e., D_2O).

To characterize the surface morphology and composition of lithium deposits, Li°||Cu° cells were discharged at a constant current density of 0.5 mA cm^{-2} for 4 hours. The cells were then disassembled to recover the Cu° substrates with deposited lithium. These electrodes were rinsed three times with diglyme to eliminate residual electrolyte, and subsequently dried under vacuum in a chamber connected to the glovebox. The surface morphology of the lithium deposits on the Cu° substrates was examined by scanning electron microscopy (SEM, Hitachi SU8100) at an accelerating voltage of 15 kV. The chemical composition of the Li° deposits was further characterized by X-ray photoelectron spectroscopy (XPS, ESCALAB 150Xi) and time-of-flight secondary ion mass spectrometry (ToF-SIMS, ToF-SIMS.5, IONTOF). To minimize possible contamination and prevent side reactions with atmospheric moisture, all samples were transferred from the glovebox to the SEM, XPS and ToF-SIMS chamber using sealed and argon-filled vessels.

1.4 Chemical Simulation

All the THF solutions containing biphenyl radical monoanions (ca. 2 wt%) were prepared by mixing biphenyl and Li° debris in THF solvent [2]. The as-prepared radical solution was gradually added into the neat NF additive, until the molar ratio of radical anion to NF reached 10:1. After stirring at 25 °C for 24 hours, the resulting mixtures were subjected to vacuum treatment to remove THF solvent, affording brownish solid products derived from the reductive decompositions of NF in the presence of an excess amount biphenyl radical monoanions. The brownish solid products were subsequently extracted with diglyme. Following filtration, the diglyme-soluble phase was transferred into coaxial NMR tubes (inner tube filled with acetone-d_6), and the chemical structures were analyzed using an NMR spectrometer (AV400, Bruker, Germany). In parallel, the diglyme-nonsoluble solid products were further analyzed by XPS.

1.5 Cell Assembly and Electrochemical Measurements

Electrochemical Li plating/stripping tests of the as-prepared electrolytes were carried out using CR2032 coin cells and a battery testing system (NEWARE CT-4008Tn) at room temperature. All the cells were assembled in an argon-filled glovebox with oxygen and water content maintained below 0.01 ppm. The Li°||Cu° coin cells consisted of a lithium metal disk and Cu foil, separated by a polypropylene membrane (Celgard 2400). For each cell, 60 μL of electrolyte was used.

The Coulombic Efficiency (CE) was evaluated using the short-term Aurbach test protocol (*cf.* Supplementary Note #1) and long-term cycling tests. For long-term cycling tests, the Li°||Cu° cells were first galvanostatically

discharged at a current density of 0.5 mA cm^{-2} for 2 hours, followed by charging at the same current density until a cut-off voltage of 1.0 V was reached. These discharge/charge (plating/stripping) cycles were repeated continuously 100 times without any rest period. All the Li°||Cu° cells were fabricated in a single batch using identical materials, including cell casings, copper foils, lithium metal chips and separators. In addition, all assembly conditions (e.g., electrolyte volume, assembly procedure, and electrochemical testing parameters) were kept strictly consistent to ensure reproducibility.

The Cu°||$LiFePO_4$ cells were assembled in CR2032 coin cells using Cu foil as the anode current collector and $LiFePO_4$ as the cathode. Cell assembly was carried out in an Ar-filled glovebox (H_2O and O_2 < 0.01 ppm) using 120 μL of electrolyte and a polypropylene membrane (Celgard 2400) separator. Galvanostatic charge/discharge measurements were conducted on LAND battery cycler at room temperature within a voltage range of 2.5–3.7 V. The cells were initially cycled at 0.1 C for the formation cycle, followed by cycling at 0.2/0.2 C for subsequent cycles. For each electrolyte formulation, four independent cells were tested in parallel. The reported capacities and Coulombic efficiencies correspond to the arithmetic mean values of the four cells, and the standard deviations (SD) were used to evaluate cell-to-cell variations.

1.6 Computational Details for Density Functional Theory Calculation

Initial geometries for all density functional theory (DFT) calculations were generated using a hierarchical and physically informed protocol. For the isolated molecular species investigated in this work, namely the $TFSI^-$ and FSI^- anions, NF additives, and DME solvent molecules, three-dimensional structures were first constructed and pre-optimized using the Universal Force Field (UFF) combined with a genetic algorithm conformational search, as implemented in the *Avogadro* molecular editor [3]. This procedure ensured efficient sampling of low-energy conformers while providing reliable starting geometries for subsequent quantum chemical refinement.

For lithium-coordinated solvation complexes, which exhibit significant conformational flexibility (e.g., $Li[DME]_3$, $Li[anion][DME]_2$, and $Li[NF][DME]_2$), a different strategy was adopted to obtain structurally realistic models. Instead of relying exclusively on static model construction, representative configurations were extracted directly from equilibrated molecular dynamics (MD) simulation trajectories. These snapshots captured thermally accessible coordination environments and dynamic structural features that are difficult to reproduce using purely heuristic or static modeling approaches.

The MD-derived structures were subsequently used as initial input geometries for the DFT calculations, thereby ensuring that the quantum chemical analyses were grounded on statistically relevant and representative solvation configurations. This combined approach, force-field-assisted pre-optimization for isolated species together with trajectory-based extraction for coordinated complexes, provided a robust and physically consistent foundation for all subsequent electronic structure calculations.

High-precision geometry optimizations and electronic structure analyses were performed using the all-electron, numeric atom-centered basis set code package, *FHI-aims* (version 200112) [4]. All DFT calculations employed

WILEY-VCH

B3LYP exchange-correlation functional [5,6]. To ensure numerical accuracy and consistency across all systems, tight numerical settings together with tier 2 standard basis sets were uniformly applied for all elements (Li, C, N, O, F, and S) involved. All geometry optimizations were carried out under stringent convergence criteria. Specifically, the maximum residual force on each atom was required to be below 0.01 eV $Å^{-1}$, the self-consistent field (SCF) electron density convergence threshold was set to 10^{-4} electrons, and the total energy convergence criterion was fixed at 10^{-6} eV.

To quantitatively evaluate the interaction strength among species within the primary solvation sheath, the binding energy (E_{bind}) of each solvated complex (e.g., $Li[A][B]_n$) was calculated as the energy difference between the fully optimized complex and the sum of its isolated constituents (Equation S1):

$$E_{bind}=E_{complex}-(E_{Li^+}+E_A+nE_B) \quad \text{(Equation S1)}$$

where $E_{complex}$, E_{Li^+}, E_A, and E_B denote the single-point total energies of the optimized solvation complex, isolated Li^+ cation, component A (anion or additive), and component B (DME solvent), respectively. A more negative E_{bind} indicates stronger thermodynamic stabilization of the solvation complex.

Weak noncovalent intermolecular interactions within the bulk electrolyte matrix were examined using representative dimer models, including DME-DME, NF-NF, and DME-NF pairs. All calculations were performed using the *ORCA* 6.0 quantum chemistry program package [7]. Geometry optimizations were performed using the B97-3c composite method with tight SCF convergence criteria [8]. Subsequently, single-point energy and wavefunction calculations were computed at the B3LYP-D3(BJ) level of theory to incorporate empirical dispersion corrections [9]. The def2-TZVP basis set, together with the corresponding def2-TZVP/C auxiliary basis set, was employed. In addition, the RIJCOSX approximation was applied to improve computational efficiency in the evaluation of Coulomb and Hartree-Fock exchange integrals [10-12].

The resulting wavefunctions were used for electrostatic potential (ESP) mapping and Independent Gradient Model based on Hirshfeld partition (IGMH) analysis. These analyses were performed using the *Multiwfn* program and visualized with *VMD*, allowing clear resolution of the spatial distribution and relative strength of weak non-covalent interactions [13,14].

Furthermore, natural bond orbital (NBO) analysis was conducted using the *NBO* 7.0 program [15]. This analysis was used to quantify intermolecular charge-transfer interactions and donor-acceptor orbital interactions between molecular fragments, providing an orbital-level interpretation of weak intermolecular interactions among DME and NF species.

WILEY-VCH

1.7 Computational Details for Molecular Dynamics Simulations

Classical MD simulations were performed using the *GROMACS* 2022.1 software package [16]. To accurately describe intermolecular interactions within the electrolyte systems, the OPLS-AA force field framework was adopted. Specifically, parameters for the $TFSI^-$ anion were derived from the work of Pádua and Lopes [17], while those for the FSI^- anion were obtained from the compatible CL&P force field [18], which is specifically optimized for ionic species. Force-field topologies and parameters for DME were generated using the LigParGen web server within the OPLS-AA framework. For the NF additive, CM5 atomic charges were employed to more accurately reproduce its electrostatic potential distribution [19]. To implicitly account for electronic polarizability and to reduce artificial ion aggregation in highly concentrated ionic environments, the partial charges of Li^+, $TFSI^-$, and FSI^- were uniformly scaled by a factor of 0.8.

To systematically investigate concentration-dependent solvation behavior and spatial distribution of the additive, MD simulation boxes were constructed with NF mass fractions of 0, 1, 3, and 5 wt% for both LiTFSI- and LiFSI-based electrolyte systems. For the LiTFSI-based electrolytes, each simulation box contained 500 LiTFSI ion pairs and 4025 DME solvent molecules, together with 0, 17, 50, and 84 NF molecules to achieve NF concentrations of 0, 1, 3, and 5 wt%, respectively. For the LiFSI-based systems, each box consisted of 500 LiFSI ion pairs and 4325 DME molecules, along with 0, 16, 48, and 80 NF molecules for the corresponding NF loadings. Initial configurations with randomly distributed, non-overlapping molecules were generated using *Packmol* [20], ensuring homogeneous dispersion of all components within the periodic cubic simulation cells prior to thermodynamic equilibration.

Each initial configuration was first subjected to energy minimization using steepest descent and conjugate gradient algorithms to eliminate unfavorable steric overlaps. The systems were then equilibrated using a multistep protocol. First, a 1 ns isothermal-isobaric (NPT) equilibration was performed, followed by a simulated annealing procedure in the canonical (NVT) ensemble. During annealing, the system temperature was raised to 400 K and subsequently cooled to 298 K over a period of 3 ns. Finally, a 1 ns NPT equilibration was conducted to ensure full thermodynamic convergence.

Production simulations were performed in the NPT ensemble for 10 ns at 298 K and 1.01325 bar to collect statistical sampling data. Temperature and pressure were controlled using the V-rescale thermostat and C-rescale barostat, respectively. The equations of motion were integrated using the leap-frog algorithm with a 2-fs time step, and all covalent bonds involving hydrogen atoms were constrained using the LINCS algorithm. Long-range electrostatic interactions were computed using the Particle Mesh Ewald (PME) method with a real-space cut-off of 1.0 nm, while van der Waals interactions were truncated at 1.0 nm. Standard analytical long-range dispersion corrections were applied to both energy and pressure. System configurations were recorded every 1 ps for subsequent trajectory analysis.

WILEY-VCH

2. Supplementary Note

2.1 Introduction to the Aurbach Test for Li°||Cu° Cell (Supplementary Note #1)

The Aurbach test is an electrochemical method used to quantify CE. A Li°||Cu° cell is assembled, and the test consists of several key stages:

Stage 0 → I (Pre-conditioning cycle): A high-capacity lithium plating/striping cycle is performed to form a stable SEI on the Cu substrate and eliminate substrate effects. First, lithium is deposited onto the Cu substrate at a current density of 0.5 mA cm^{-2} with an areal capacity of 5 mAh cm^{-2}.Then, the deposited lithium is fully stripped to an upper cut-off voltage of 1 V (vs. Li^+/Li). This conditioning cycle is not included in the final CE calculation.

Stage I → II: A specific amount of lithium (Q_1 = 5 mAh cm^{-2}: deposited at 0.5 mA cm^{-2} for 10 h) is deposited onto the conditioned Cu substrate, transforming the cell into a symmetric Li°||Li@Cu configuration. The open-circuit voltage (ca. 3 V) drops to approximately −50 mV, depending on the electrolyte.

Stage II → III: Repeated stripping and plating cycles are performed with a fixed, lower capacity. Each half-cycle (plating or stripping) is carried out at a current density of 0.5 mA cm^{-2} for 2 hours (Q_2 = 1 mAh cm^{-2}) If the deposited lithium is sufficiently retained, the voltage profiles remain low (tens to hundreds of millivolts) due to the symmetric cell setup.

Stage III →IV: After *n* cycles, the remaining lithium is exhaustively stripped back to the Li° electrode at the cut-off voltage of 1 V, and the stripping capacity is recorded as Q_3.

The irreversible loss of active lithium over *n* cycles is calculated using Equation S2:

$$CE_{\text{Aurbach test}}=\frac{nQ_2+Q_3}{nQ_2+Q_1} \quad \text{(Equation S2)}$$

2.2 Concentration-Independent Solvation Architecture in the LiTFSI-Based Electrolyte (Supplementary Note #2)

To systematically evaluate the influence of NF additive concentration on the intrinsic solvation structure, molecular dynamics (MD) simulations were performed across a concentration gradient of 0, 1, 3, and 5 wt% NF in the LiTFSI-based electrolyte. As depicted in the radial distribution function (RDF) and coordination number (CN) profiles (Figure S5), the primary solvation shell of Li^+ exhibits high structural rigidity and is largely insensitive to variations in NF content.

Across all investigated concentrations, the Li-O_{DME} coordination pair consistently displays a sharp and intense peak at an interatomic distance of approximately 2.08 Å. Quantitative integration of these peaks reveals that the

CN (ca. 6.0) of Li-O_{DME} remains highly stable for LiTFSI-based electrolyte with varying amounts of NF, confirming that the characteristic six-fold coordination of Li^+ by DME molecules is maintained. Concurrently, Li-TFSI coordination within the primary solvation sheath remains negligible across all concentrations, indicating that the bulky $TFSI^-$ anions are consistently excluded from the inner solvation shell regardless of additive mass fraction. Most importantly, the CNs for all possible Li-NF interaction pairs, including Li-O_{NF}, Li-F_{NF_CF}, and Li-F_{NF_SF}, remain zero from 1 to 5 wt% NF, which suggests that NF molecules do not participate in the inner coordination sphere of Li^+.

Furthermore, analysis of the local microenvironment surrounding NF molecules reveals that the additive remains highly dispersed throughout the bulk electrolyte matrix. As the NF concentration increases from 1 to 5 wt%, the immediate coordination environment of NF is persistently dominated by DME, with a steady average of approximately 8 to 9 DME molecules within a 3.0 Å radius. Although the average number of neighboring NF molecules around a central NF molecule increases slightly with additive concentration, it remains extremely low, rising from nearly 0 at 1 wt% to approximately 0.2 at 5 wt% (Figure S12). This marginal change demonstrates that NF does not form significant local clusters under current calculation conditions.

Similarly, analysis of the local microenvironment surrounding $TFSI^-$ anions reveals a consistent structural pattern. As the NF concentration increases from 1 to 5 wt%, the immediate coordination vicinity of $TFSI^-$ remains dominated by DME, with a highly stable average of approximately 10 to 11 DME molecules within a 3.0 Å radius. Although the average number of neighboring NF molecules around a central $TFSI^-$ anion increases with additive content, it remains very low, increasing from nearly zero at 1 wt% to approximately 0.15 at 5 wt% (Figure S14). Notably, this subtle yet systematic enrichment of NF in the local environment provides a molecular-level explanation for the observed chemical shift variations of the CF_3– group of $TFSI^-$ in the macroscopic ^{19}F NMR spectra (Figure 2G).

In summary, within the current computational parameter setup, these statistical analyses demonstrate that the fundamental solvent-separated ion pair (SSIP) architecture of the LiTFSI-based electrolyte is intrinsically robust. The introduced NF additive is accommodated within the interstitial regions of the DME network, functioning as a fully decoupled, non-coordinating species that preserves the primary solvation environment of Li^+.

2.3 Concentration-Independent Solvation Architecture in the LiFSI-Based Electrolyte (Supplementary Note #3)

To systematically evaluate the effect of NF additive concentration on the intrinsic solvation structure of the LiFSI-based electrolyte, MD simulations were conducted across an NF concentration range of 0, 1, 3, and 5 wt%. As depicted in the RDF and CN profiles (Figure S6), the primary solvation shell of Li^+ remains essentially unchanged with increasing NF content.

For all investigated NF concentrations, the Li-O_{DME} and Li-O_{FSI} coordination pairs consistently display sharp, intense RDF peaks at approximately 2.08 Å. Integration of these peaks reveals that the corresponding CNs for Li-

WILEY-VCH

O_{DME} and Li-O_{FSI} remain essentially invariant, fluctuating only negligibly around 3.9 and 1.6, respectively, over the entire 0-5 wt% NF range. This confirms that the typical contact-ion-pair (CIP) configuration (i.e., Li[FSI][DME]$_2$) is preserved throughout all concentrations. The persistent Li^+-FSI coordination further indicates that the FSI^- anion remains firmly anchored within the primary solvation sheath regardless of NF loading. Importantly, the CNs for all possible Li-NF interaction pairs, including Li-O_{NF}, Li-F_{NF_CF}, and Li-F_{NF_SF}, remain zero from 1 to 5 wt% NF, which suggests that NF molecules do not participate in the inner coordination sphere of Li^+.

Analysis of the local environment around NF molecules further reveals that the additive remains highly dispersed within the bulk electrolyte. As the NF concentration increases from 1 to 5 wt%, the immediate NF-centered microenvironment remains dominated by DME, with an average of approximately 8.5 to 9.0 DME molecules within a 3.0 Å radius. Although the number of neighboring NF molecules around a central NF molecule increases slightly with loading, it remains extremely low, rising from nearly 0 at 1 wt% to only 0.2 at 5 wt% (Figure S12). This negligible increase demonstrates that NF does not form significant local clusters under current calculation conditions.

A similar structural trend is observed for FSI^- anions. From 1 to 5 wt% NF, the FSI-centered local environment remains dominated by DME, with a stable average of approximately 8 DME molecules within a 3.0 Å radius, while maintaining coordination with ca. 1.1 Li^+ cations. The number of NF in the vicinity of a central FSI^- anion increases only marginally with concentration, rising from nearly zero at 1 wt% to approximately 0.10 at 5 wt% (Figure S14). Importantly, this subtle yet systematic increase of NF around FSI^- provides a molecular-level explanation for the concentration-dependent chemical shift variations observed in the macroscopic ^{19}F NMR spectra (Figure 2G).

In summary, within the current computational parameter setup, these statistical analyses show that the fundamental CIP solvation architecture of the LiFSI-based electrolyte is intrinsically robust and largely insensitive to NF concentration. Rather than entering the Li^+ primary solvation shell, NF occupies interstitial spaces within the DME-rich electrolyte network. Consequently, NF acts as a non-coordinating, structurally decoupled additive that preserves the native Li^+ solvation environment while subtly modulating the local chemical environment of FSI^- anions.

WILEY-VCH

2.4 Thermodynamic and Quantum Chemical Origins of the NF-DME Phase Separation (Supplementary Note #4)

To elucidate the fundamental origin of the macroscopic liquid-liquid phase separation observed when NF is directly dispersed in pure DME, we performed a theoretical analysis based on density functional theory (DFT). The tendency toward phase separation is fundamentally dictated by the pronounced disparity in intermolecular binding energies (Figure S8). The intrinsic DME-DME interaction yields a stabilization energy of -1.42 kJ mol^{-1}, which is significantly stronger than the cross-interaction between DME and NF, i.e., DME-NF interaction (-0.22 kJ mol^{-1}). In contrast, NF-NF interactions are slightly repulsive, with a binding energy of $+0.04$ kJ mol^{-1}. Consequently, the strong cohesive interactions within the DME solvent network thermodynamically favor self-association, whereas the weak and energetically unfavorable NF interactions within the DME-rich phase led to its exclusion from the bulk solvent environment.

This substantial difference in intermolecular affinity originates from the distinct electronic structures of DME and NF, as revealed by electrostatic potential (ESP) maps (Figure S9). DME molecules exhibit pronounced electrostatic polarization, with clearly defined electron-rich and electron-deficient regions that readily promote strong dipole-dipole and donor-acceptor interactions. In contrast, the perfluorinated NF molecule presents a comparatively uniform and weakly polarized electrostatic surface. This electronically inert character limits its ability to form strong electrostatic interactions with either the polar DME molecules or neighboring NF molecules.

To further quantify the spatial characteristics of these noncovalent interactions, Independent Gradient Model based on Hirshfeld partition (IGMH) analysis was conducted (Figure S10). The integrated intermolecular δg index is highest for the DME-DME dimer (0.110970 a.u.), indicating strong cohesive interactions within the pure DME network. The corresponding values decrease markedly for the DME-NF and NF-NF dimers, to 0.081819 and 0.056524 a.u., respectively. This trend confirms that the intermolecular interactions involving NF are significantly weaker than the intrinsic DME-DME cohesion, providing a spatially resolved explanation for the poor miscibility of NF in neat DME.

Finally, Natural Bond Orbital (NBO) second-order perturbation theory analysis (Figure S11) elucidates the origin of this interaction hierarchy at the orbital level. In the DME-DME dimer, an efficient donor-acceptor interaction arises from the oxygen lone pair of one DME molecule to the C–H antibonding orbital of a neighboring DME molecule, yielding a stabilization energy of 0.32 kcal mol^{-1}. This orbital stabilization decreases to only 0.12 kcal mol^{-1} in the DME-NF dimer, indicating a significantly weaker intermolecular charge-transfer character. For the NF-NF dimer, the intermolecular orbital interactions fall below the default extraction threshold, demonstrating the absence of meaningful charge transfer or covalent-like orbital coupling between NF molecules.

Collectively, the binding-energy comparisons, ESP analysis, IGMH visualizations, and NBO perturbation results provide a consistent molecular picture: DME molecules preferentially self-associate through stronger electrostatic and donor-acceptor interactions, whereas NF lacks sufficient electrostatic polarity and orbital-

interaction capability to integrate into the DME solvent network. This thermodynamic and electronic mismatch drives the exclusion of NF from neat DME, ultimately leading to macroscopic NF-DME liquid-liquid phase separation.

WILEY-VCH

3. Supplementary Tables

Table S1. Raman peak positions and area ratios for the LiTFSI-based electrolytes with varying NF content.

Sample	Peak position (cm^{-1})			Area ratio (%)	
	$\tilde{\nu}_1$	$\tilde{\nu}_2$	$\tilde{\nu}_3$[1]	A_1	A_2
**LTF-0N**	310.54	326.06	1244.11	49.61	50.39
**LTF-1N**	311.03	326.08	1244.11	45.38	54.62
**LTF-3N**	310.58	325.86	1244.16	46.43	53.56
**LTF-5N**	310.69	326.13	1244.10	44.01	55.99

[1] $\tilde{\nu}_3$ shows no meaningful variation (constant at 1244.1 cm^{-1}) and is listed for completeness.

WILEY-VCH

Table S2. Raman peak positions and area ratios for the LiFSI-based electrolytes with varying NF content.

Sample	Peak position (cm^{-1})		Area ratio (%)	
	$\tilde{\nu}_1$	$\tilde{\nu}_2$	A_1	A_2
**LF-0N**	1216.83	1221.05	68.28	31.72
**LF-1N**	1216.63	1221.31	66.13	33.87
**LF-3N**	1216.85	1221.23	67.49	32.51
**LF-5N**	1216.88	1221.25	69.33	30.67

To further evaluate the influence of NF on the anion solvation structure, Raman spectra in additional frequency regions are analyzed. For LiTFSI-based electrolytes, the bands at approximately 310 and 326 cm^{-1} are assigned to the trans- and cis-$TFSI^-$ conformers, respectively. No significant variation in the relative intensity ratio of these bands is observed after NF addition. Furthermore, the $–SO_2–$ stretching band at approximately 1244 cm^{-1} exhibits negligible changes in both peak position and intensity. For LiFSI-based electrolytes, the fitting results in the 1200–1250 cm^{-1} region show nearly unchanged peak positions and relative intensity ratios upon NF addition. These results are consistent with the analysis of the 680–760 cm^{-1} region, indicating that NF does not significantly alter the anion solvation structure.

WILEY-VCH

Table S3. XPS characteristic peaks and chemical assignments of SEI components as reported in literature.

Species	Binding energy (eV)				
	Li 1s	C 1s	F 1s	S 2p	Ref
Li	52.2				[21]
LiF	55.5–56.0		684.8–685.2		[22, 23]
–CF_x		292.8	687.0–689.5		[24, 25]
–SO_xF			688.5	169.1–170.2	[26, 27]
S(VI) (e.g., –S(=O)$_2$–)				169.0–170.2	[22, 27]
S(IV) (e.g., –S(=O)–)				167.0–168.1	[26]
S_x^{2-}				162.9–164.8	[22]

WILEY-VCH

4. Supplementary Figures

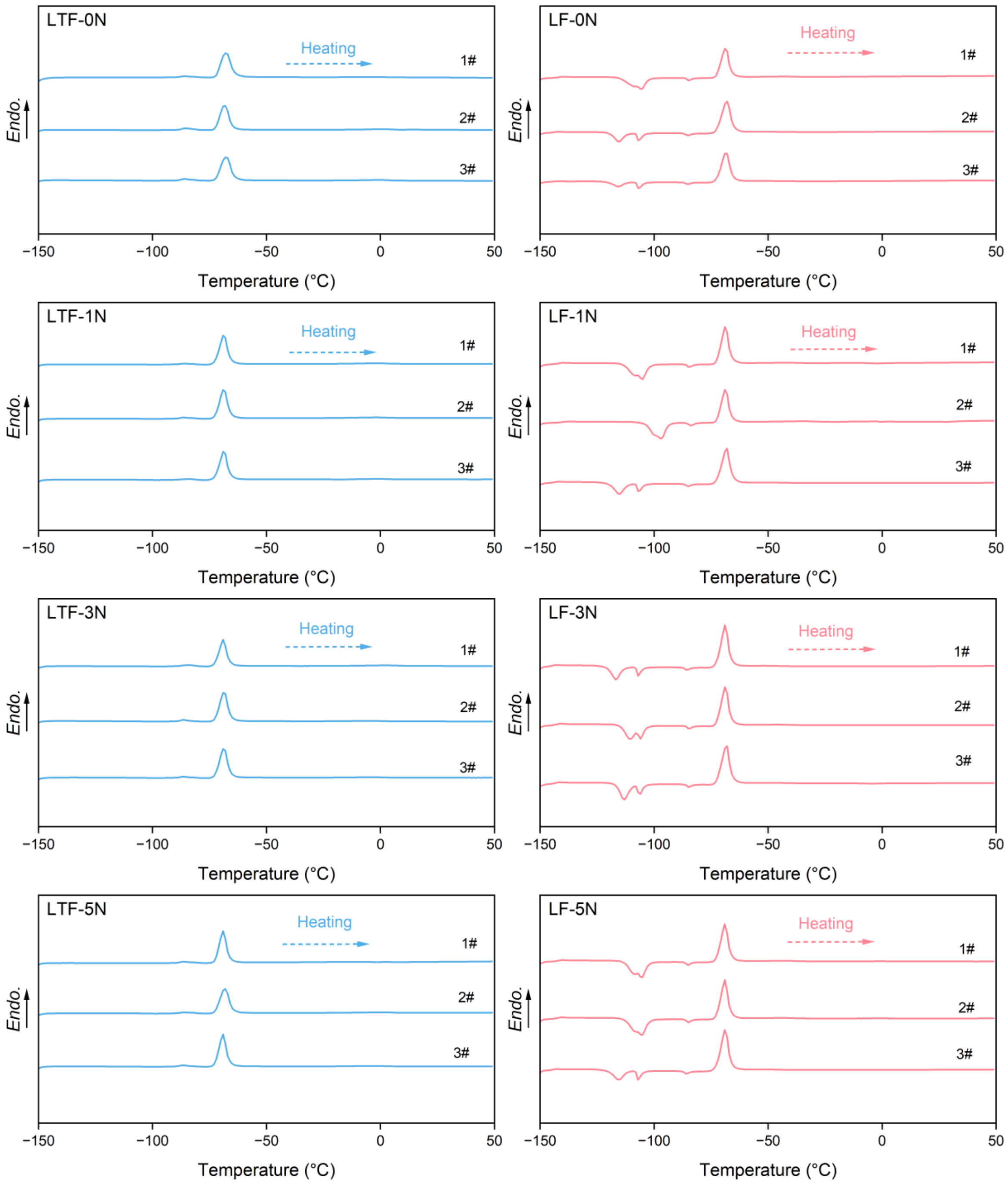

Figure S1. The full-range DSC curves of the LiTFSI- and LiFSI-based electrolytes with different NF content; three parallel samples were measured for each electrolyte.

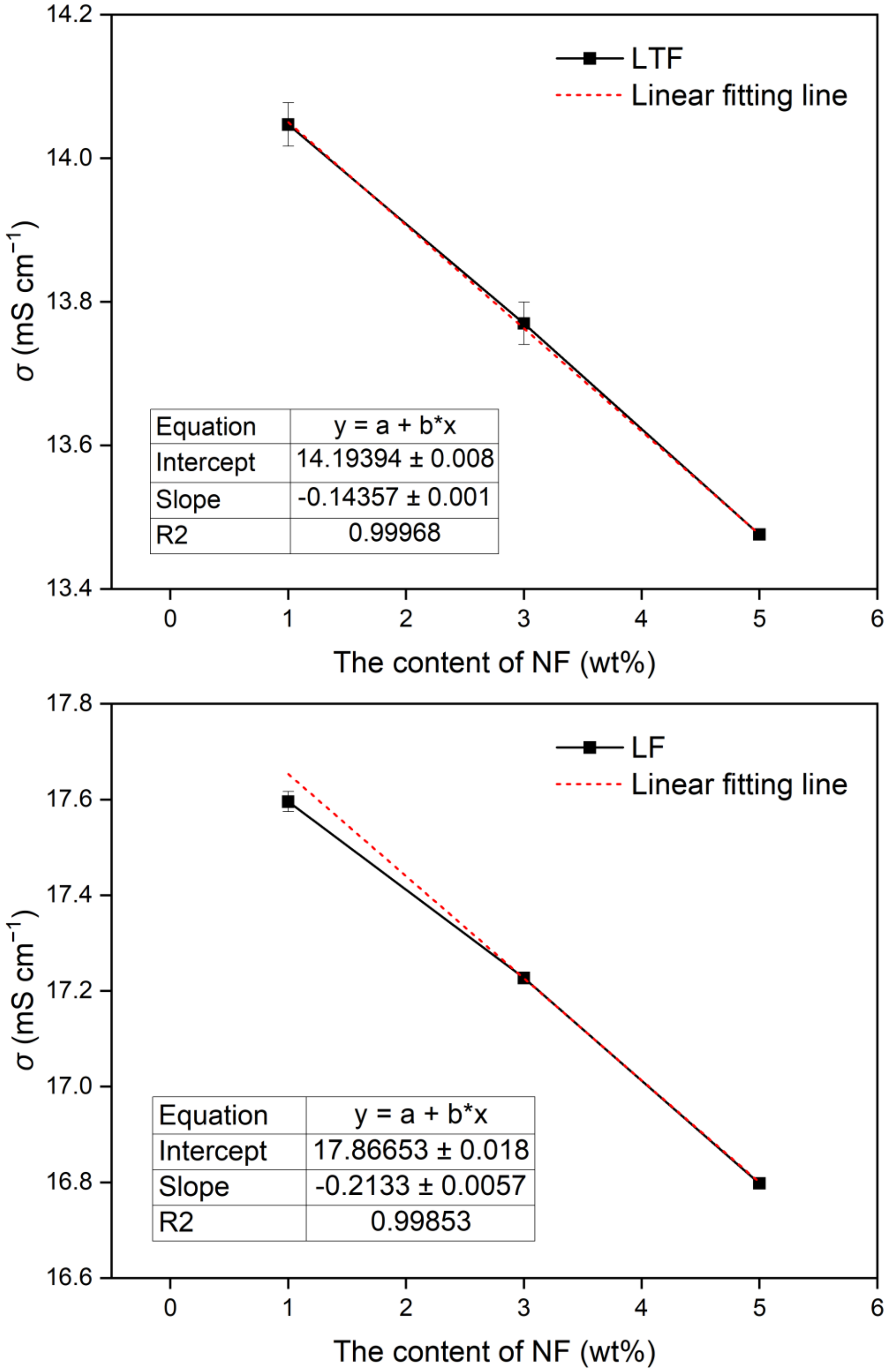

Figure S2. Linear fitting of conductivity versus NF content from 1 to 5 wt% yields similar slopes for both LiTFSI- and LiFSI-based electrolytes.

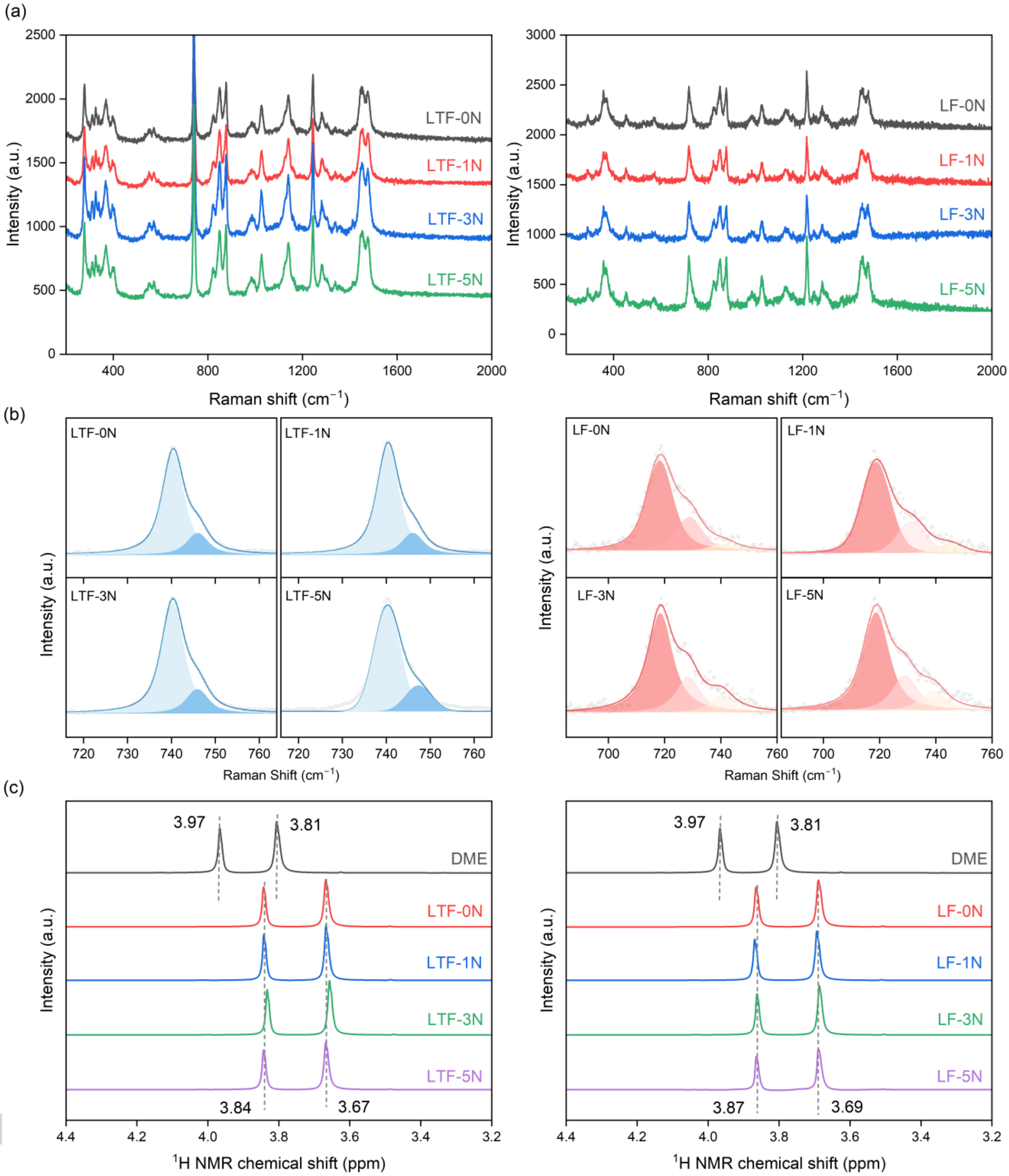

Figure S3. (a) Raman spectroscopy and corresponding high-resolution fine spectrum of the LiTFSI- and LiFSI-based electrolytes with different NF content. (b) Percentages of SSIP, CIP, AGG (aggregate) species (normalized to 100%) in different electrolytes. (c) ^{1}H NMR spectra for the LiTFSI- and LiFSI-based electrolytes with different NF contents, in which the chemical shift values at 3.97 and 3.81 ppm correspond to the methylene (–C$\underline{\boldsymbol{H}}_2OCH_3$) and methyl (–CH$_2OC\underline{\boldsymbol{H}}_3$) protons in the neat DME solvent, respectively.

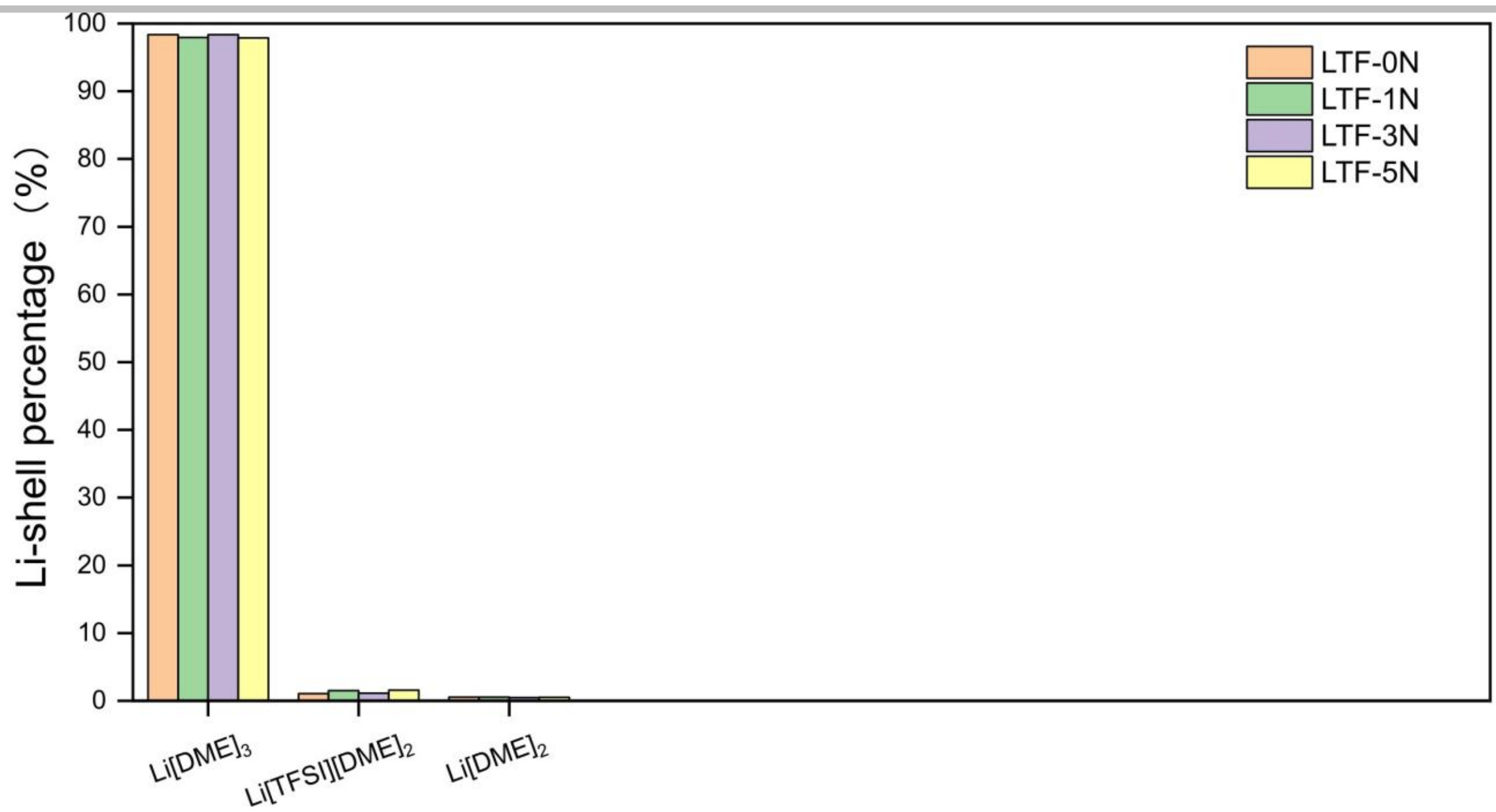

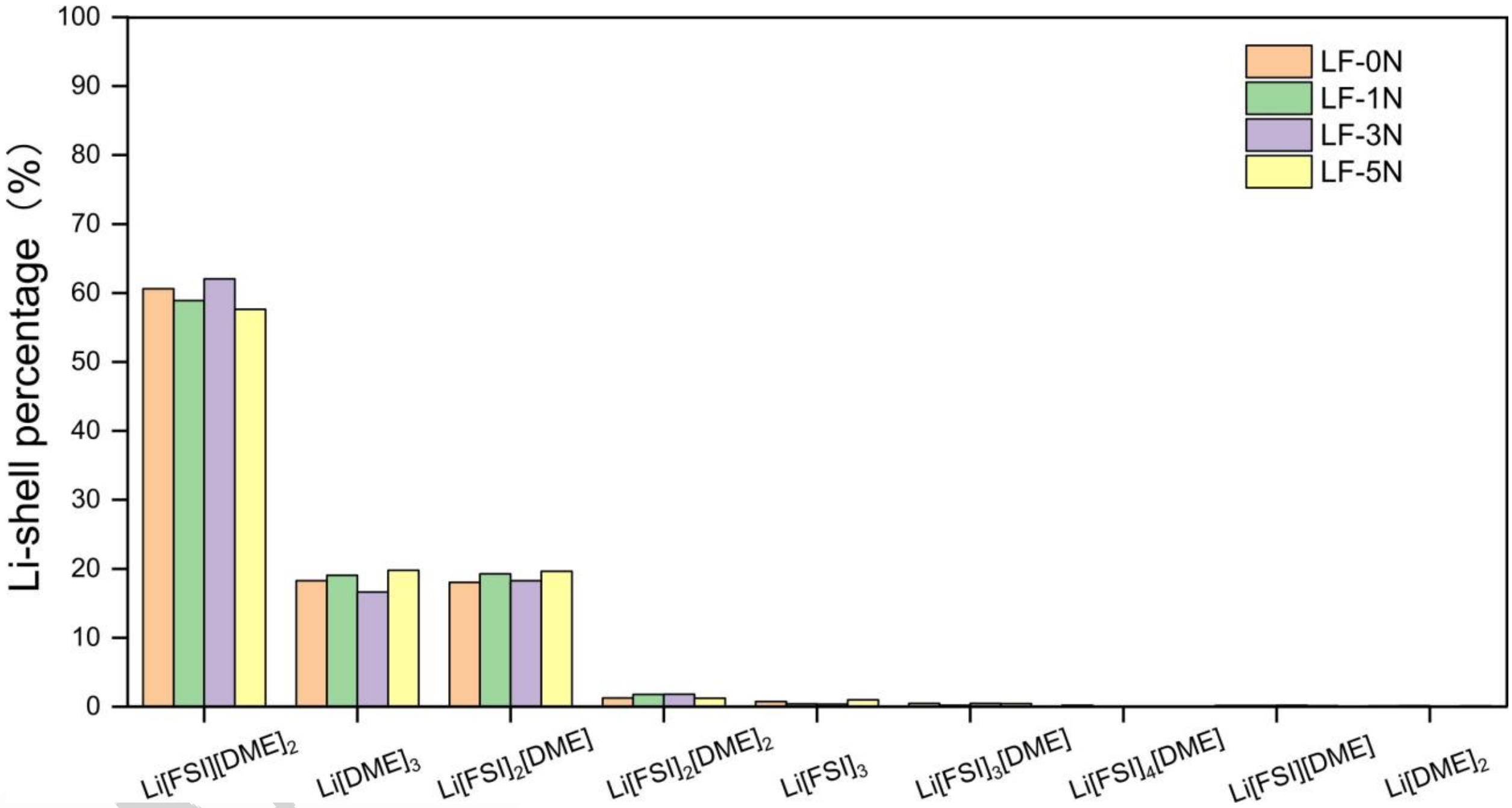

Figure S4. Statistical distribution of the Li^+ primary solvation shell configurations extracted from MD trajectories. The bar charts display the relative percentage of various solvated complexes in the LiTFSI-based electrolytes (top panel) and LiFSI-based electrolytes (bottom panel) as a function of NF concentration: 0, 1, 3, and 5 wt%. For the LiTFSI-based electrolyte, the SSIP configuration, $Li[DME]_3$, overwhelmingly dominates the primary solvation sheath at approximately 98%. In striking contrast, the LiFSI-based system is characterized by the coexistence of $Li[FSI][DME]_2$ (CIP, ca. 60%), $Li[DME]_3$ (SSIP, ca. 20%), and $Li[FSI]_2[DME]$ (AGG, ca. 20%).

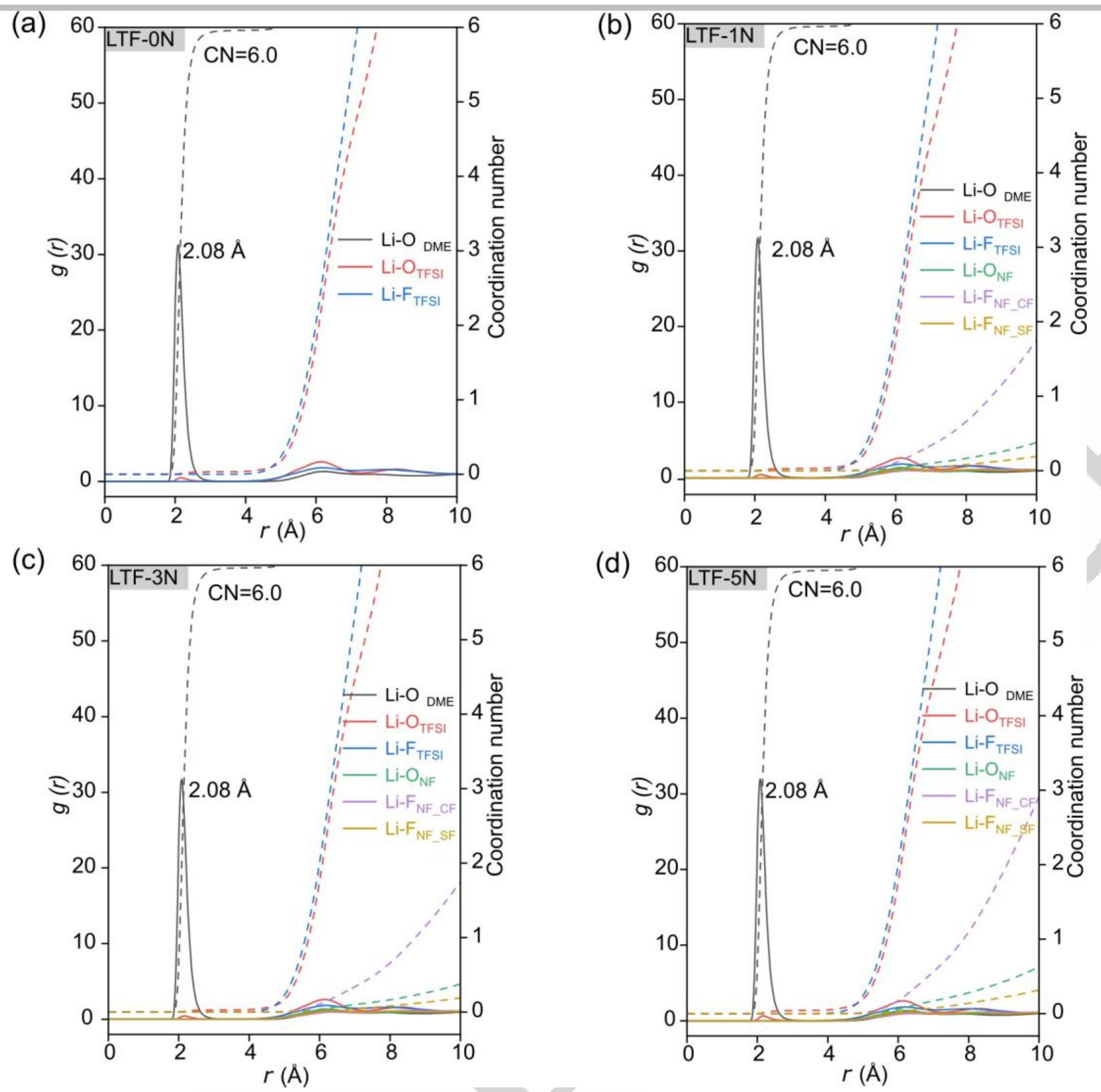

Figure S5. RDF and corresponding CN profiles centered on Li^+ extracted from the MD trajectories of LiTFSI-based electrolytes. Panels (a), (b), (c), and (d) display the local coordination environments for the systems containing 0 wt%, 1 wt%, 3 wt%, and 5 wt% NF additives, respectively.

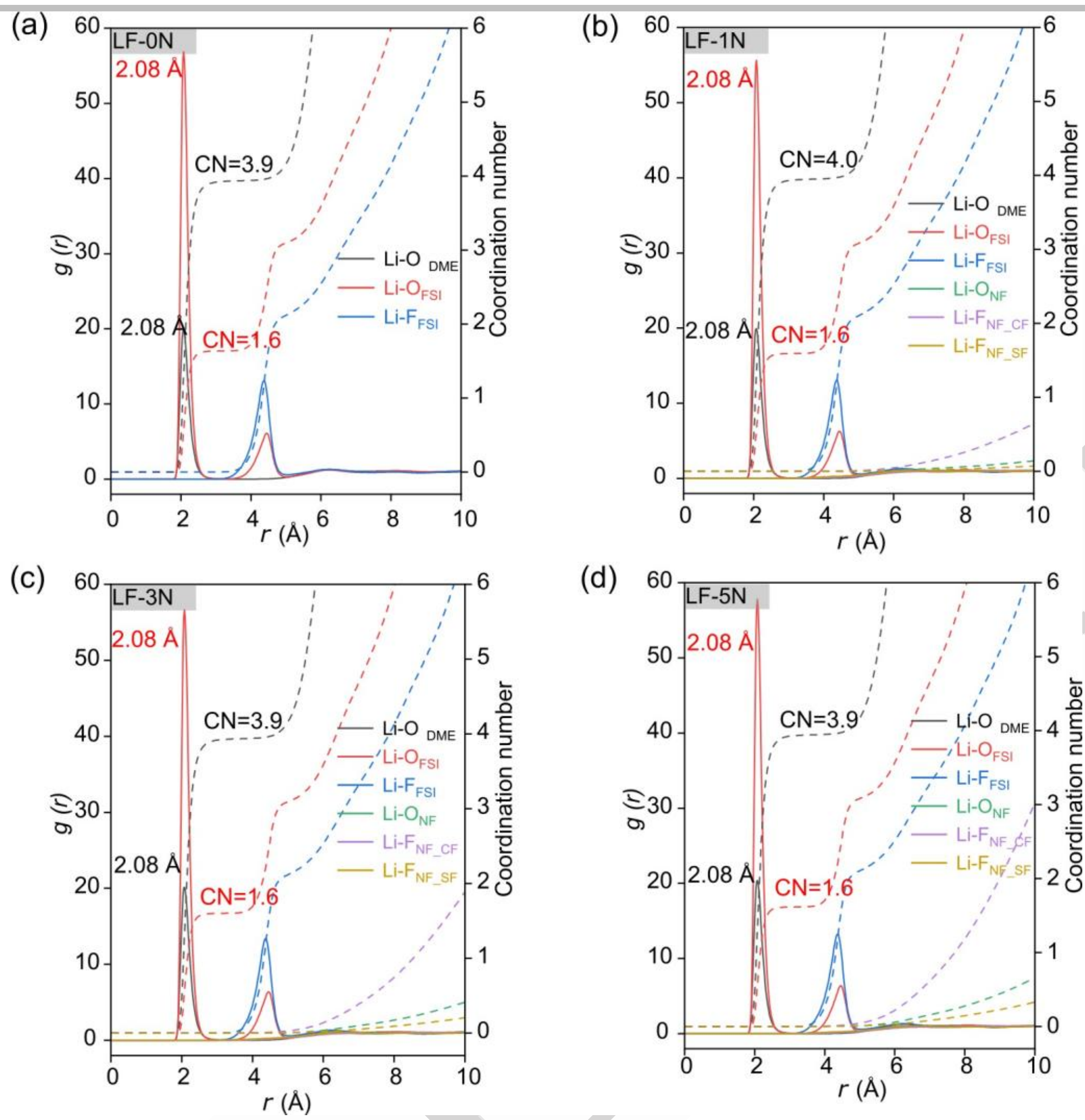

Figure S6. RDF and corresponding CN profiles centered on Li^+ extracted from the MD trajectories of LiFSI-based electrolytes. Panels (a), (b), (c), and (d) display the local coordination environments for the systems containing 0 wt%, 1 wt%, 3 wt%, and 5 wt% NF additives, respectively.

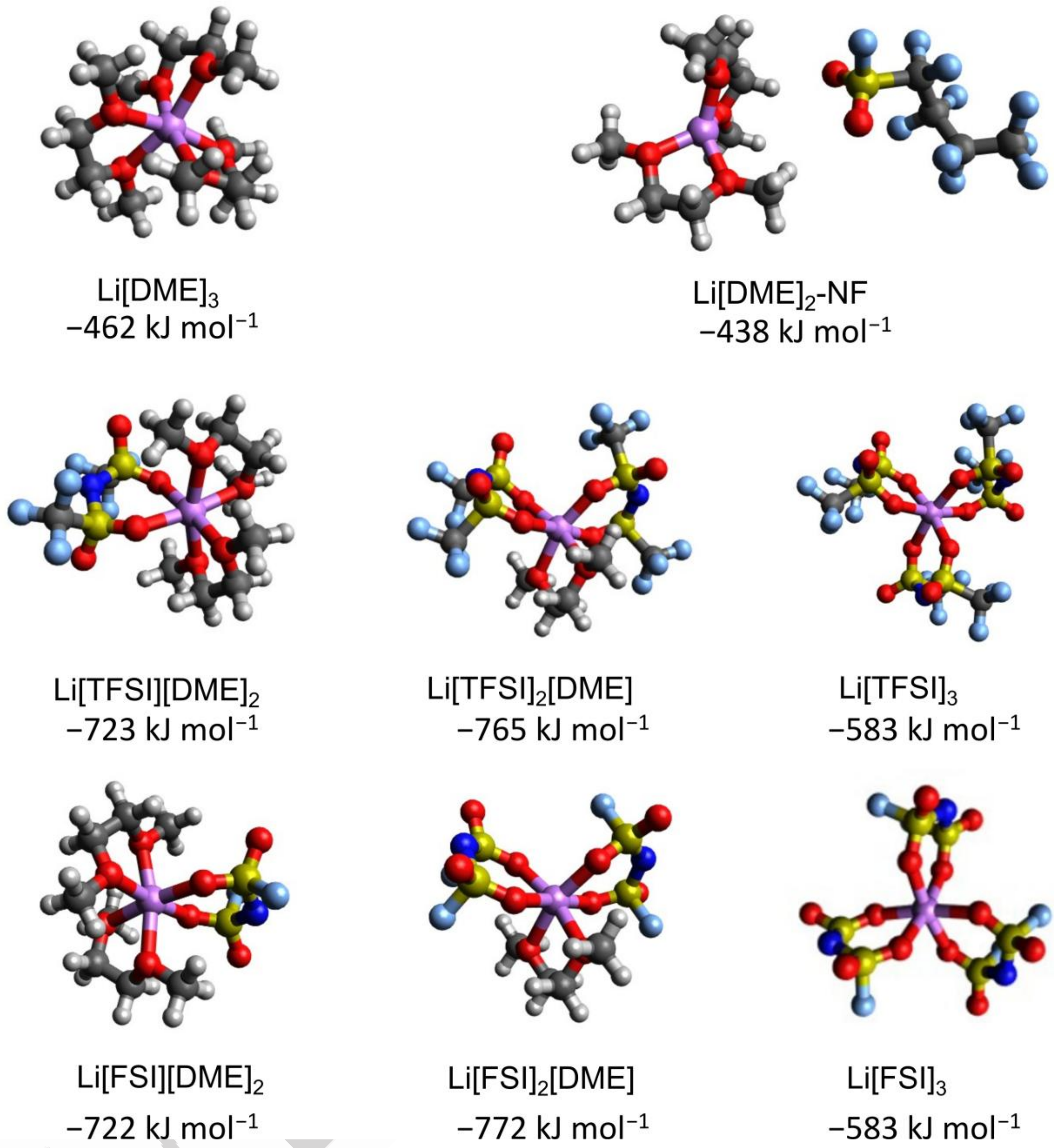

Figure S7. Optimized geometric structures and corresponding DFT binding energies of various Li^+ solvation complexes. The top row presents the baseline pure solvent complex $Li[DME]_3$ (−462 kJ mol^{-1}) alongside the artificially constructed NF-participating complex $Li[DME]_2[NF]$ (−438 kJ mol^{-1}). The middle and bottom rows display the coordination complexes featuring varying stoichiometric ratios of $TFSI^-$ and FSI^- anions, respectively, including the $Li[anion][DME]_2$, $Li[anion]_2[DME]$, and $Li[anion]_3$ configurations.

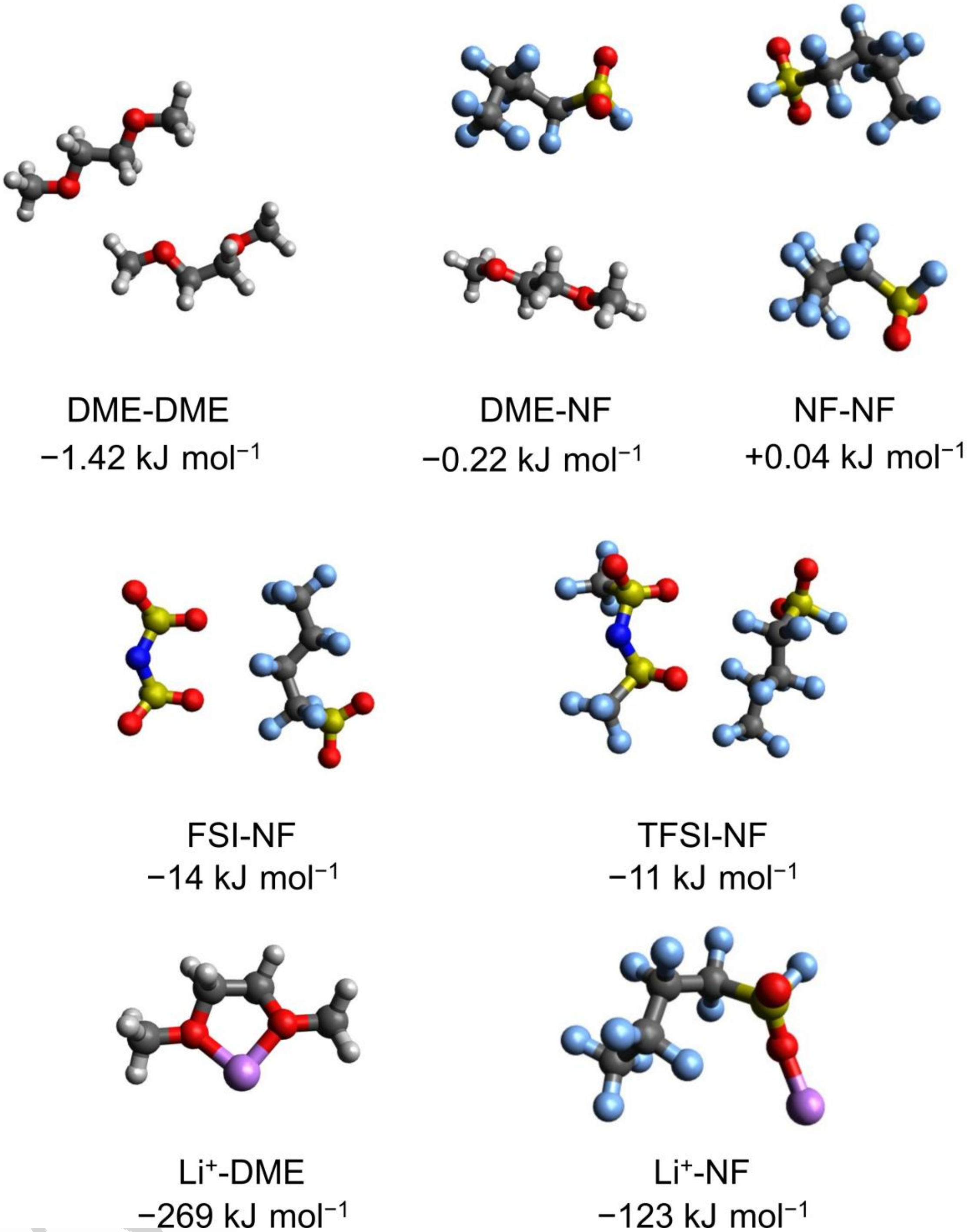

Figure S8. DFT binding energies of representative molecular dimers and ion-molecule pairs.

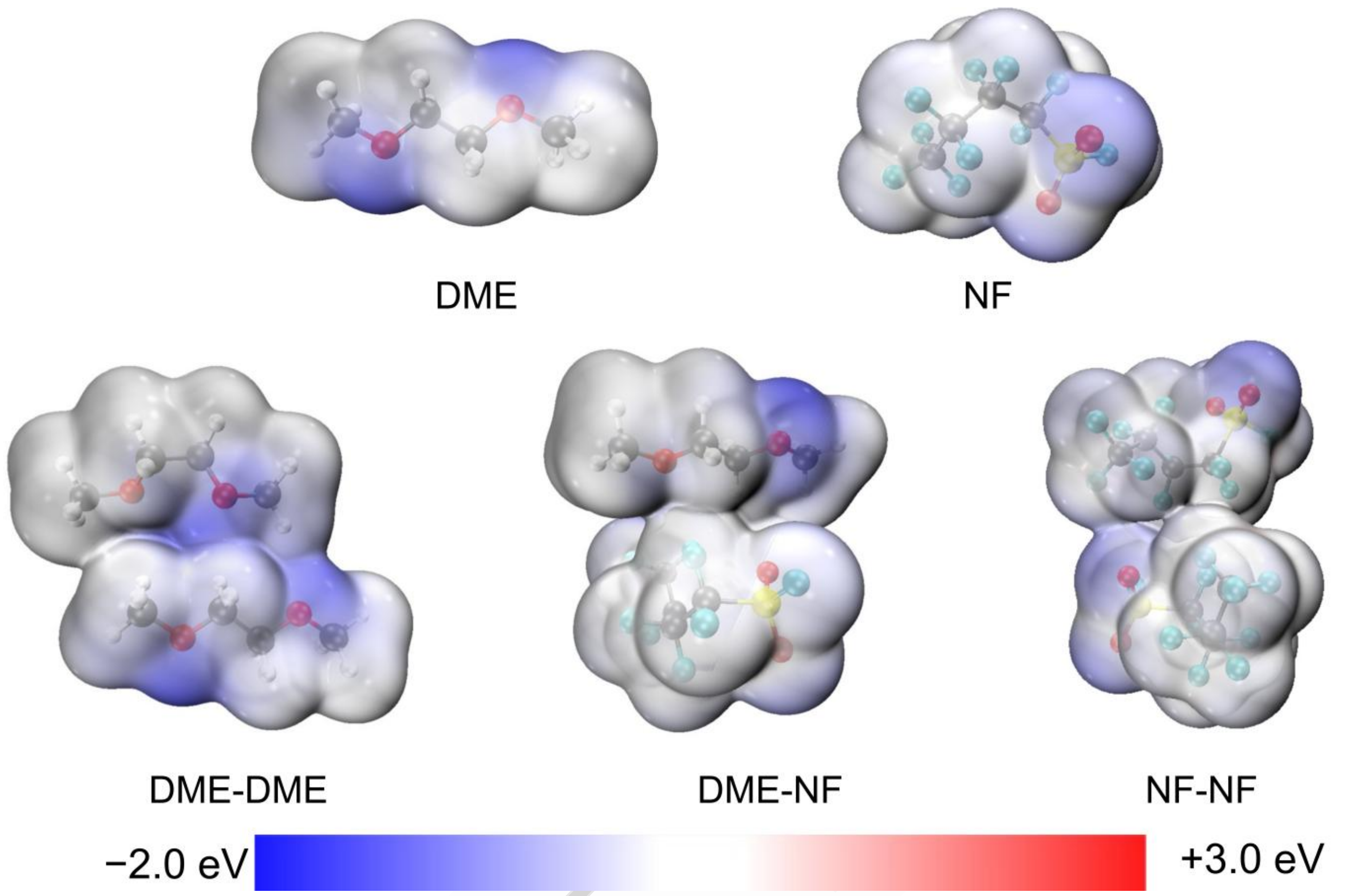

Figure S9. ESP maps of isolated molecules and their representative interacting dimers calculated to elucidate weak intermolecular interactions. The top row presents the ESP distributions mapped onto the molecular van der Waals surfaces of an individual DME solvent molecule (left) and an NF additive molecule (right). The bottom row illustrates the ESP mappings for the optimized weakly interacting dimer models: DME-DME (left), DME-NF (middle), and NF-NF (right). The color scale ranges from −2.0 eV (blue) to +3.0 eV (red), visualizing localized electron-rich and electron-deficient regions, respectively.

WILEY

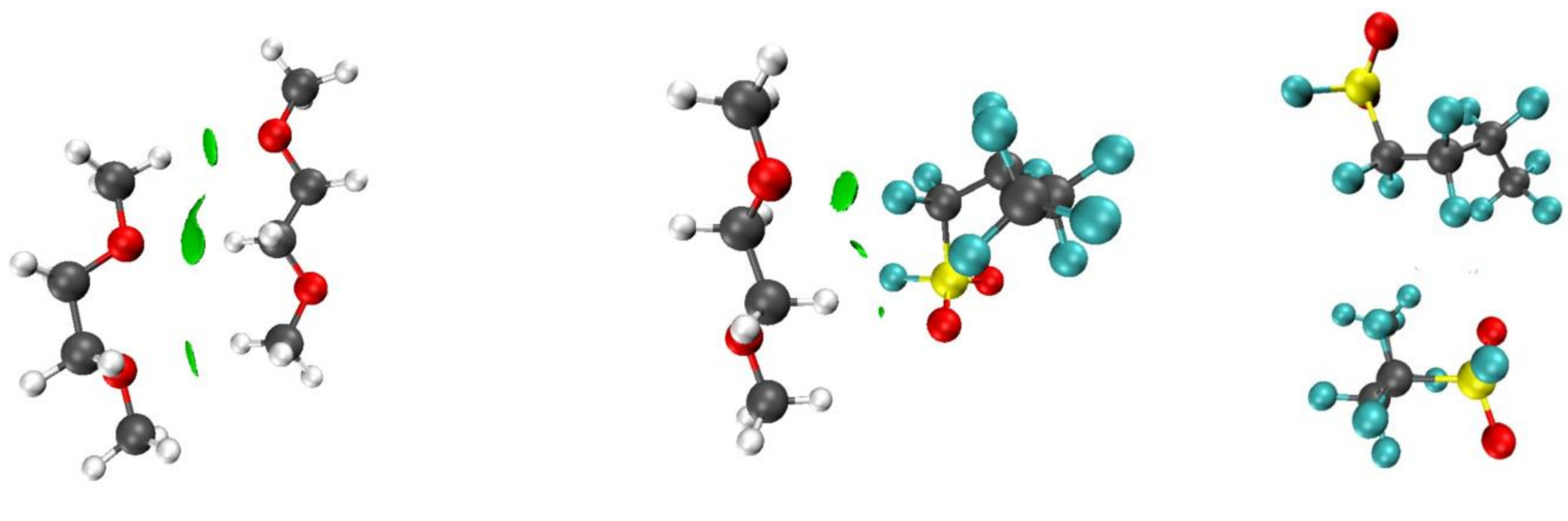

0.110970 a.u. 0.081819 a.u. 0.056524 a.u.

Figure S10. IGMH analysis illustrating the weak non-covalent intermolecular interactions within representative dimer models. From left to right, the visualized structures correspond to the DME-DME, DME-NF, and NF-NF interacting dimers, respectively. The green isosurfaces visually map the regions of spatial van der Waals and weak electrostatic interactions between the molecular fragments. To quantitatively evaluate the interaction strength, the integral of the intermolecular δg index over the whole space was extracted.

WILEY-VCH

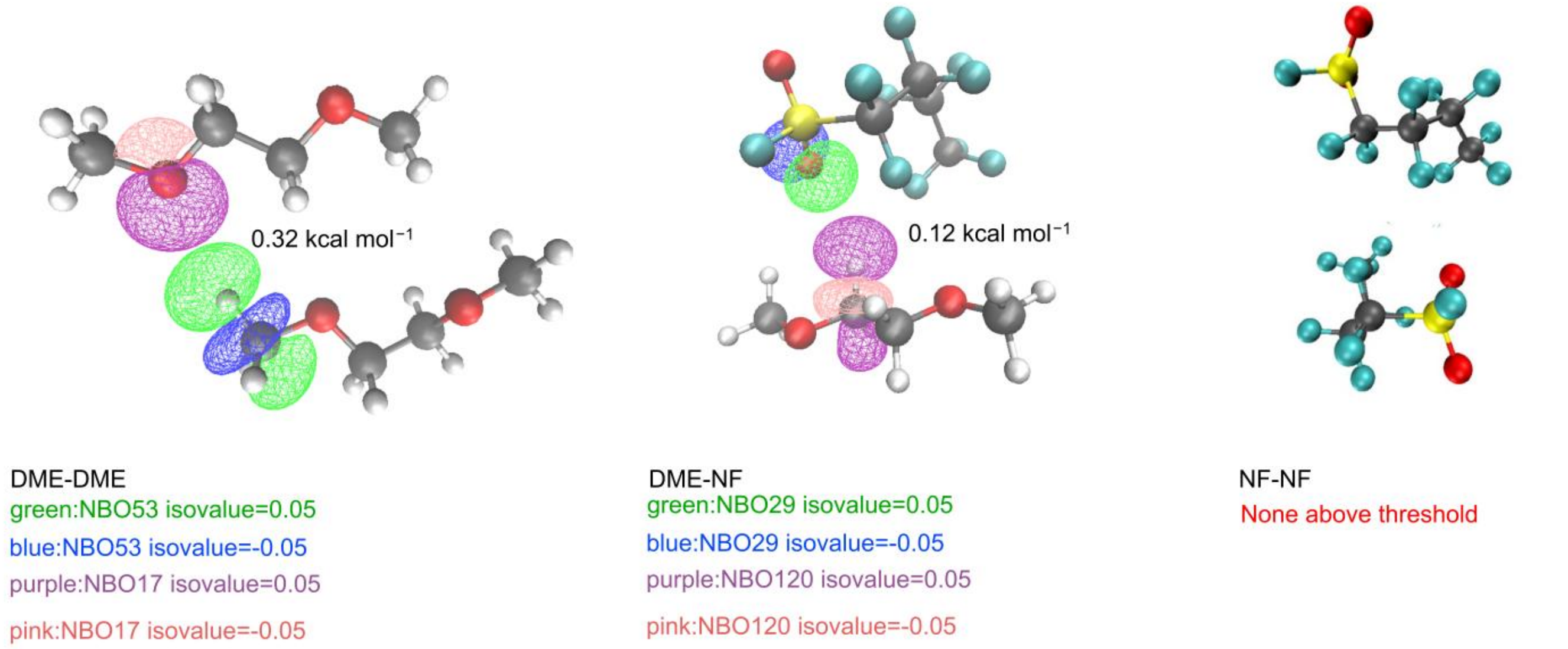

Figure S11. NBO second-order perturbation theory analysis elucidating the intermolecular donor-acceptor interactions within representative dimer models. From left to right, the molecular structures and corresponding extracted data tables represent DME-DME, DME-NF, and NF-NF interacting dimers, respectively. To quantitatively evaluate the interaction strength, the stabilization energy is also provided.

WILEY

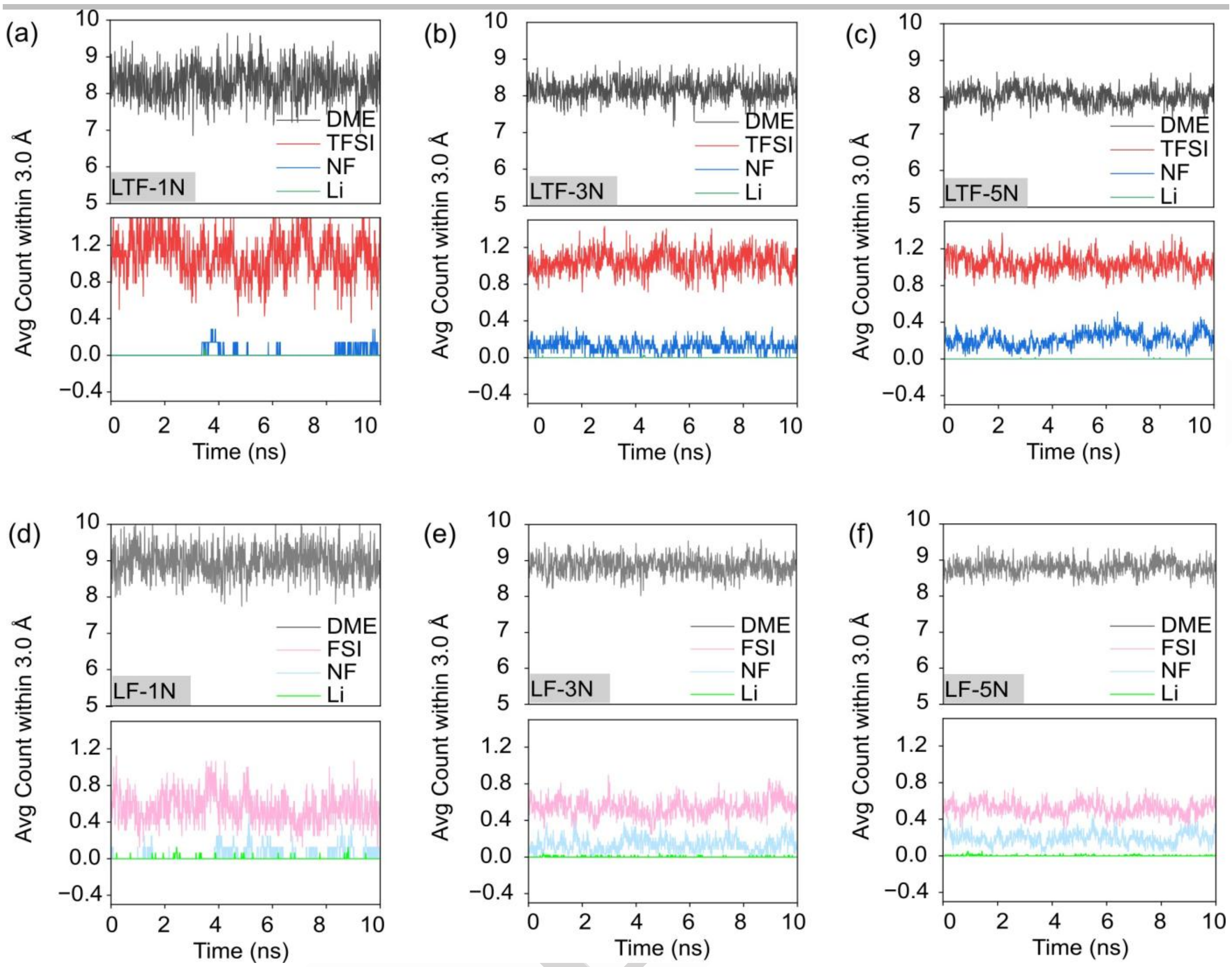

Figure S12. Evolution of the local solvation environment around the NF additive extracted from the 10 ns production MD trajectories after equilibration. The plots display the time-dependent average count of DME, anions ($TFSI^-$ or FSI^-), NF, and Li^+ within a 3.0 Å cut-off radius centered on an individual NF molecule. Panels (a-c) present the local coordination environments in the LiTFSI-based electrolytes with 1 wt%, 3 wt%, and 5 wt% NF, respectively. Panels (d-f) display the corresponding environments in the LiFSI-based electrolytes with 1 wt%, 3 wt%, and 5 wt% NF.

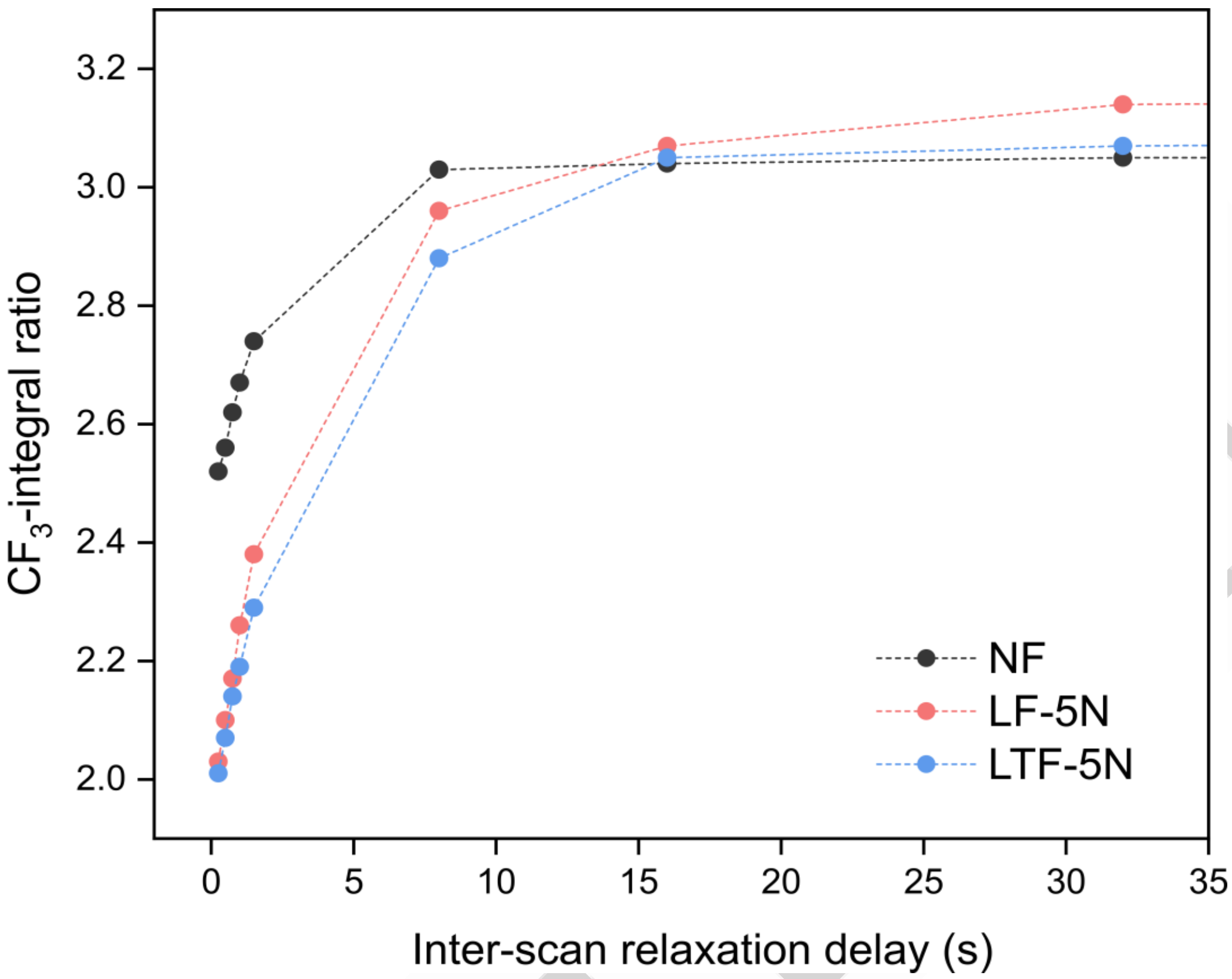

Figure S13. ^{19}F NMR longitudinal relaxation measurements. The plot displays the inversion recovery curves, showing the CF_3 integral ratio as a function of the relaxation delay time (D1) for the pure NF system, the LiFSI-based electrolyte, and the LiTFSI-based electrolyte.

WILEY-VCH

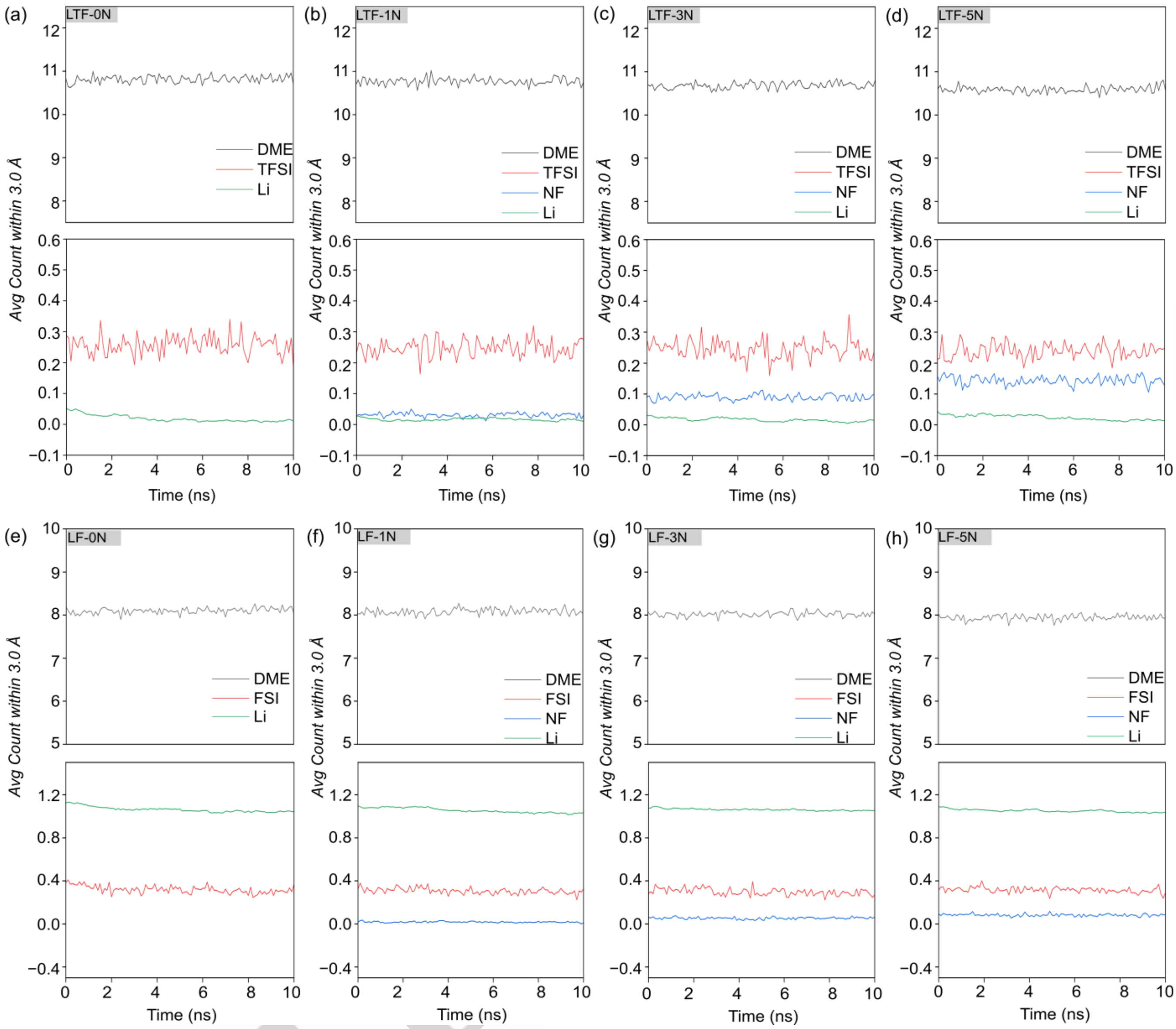

Figure S14. Evolution of the local solvation environment centered on the salt anions extracted from 10 ns equilibrated MD simulations. The plots display the time-dependent average count of DME, anions, NF, and Li^+ within a 3.0 Å cut-off radius centered on an individual $TFSI^-$ anion (panels a-d) and an individual FSI^- anion (panels e-h) with varying NF additive concentrations (0, 1, 3, and 5 wt%).

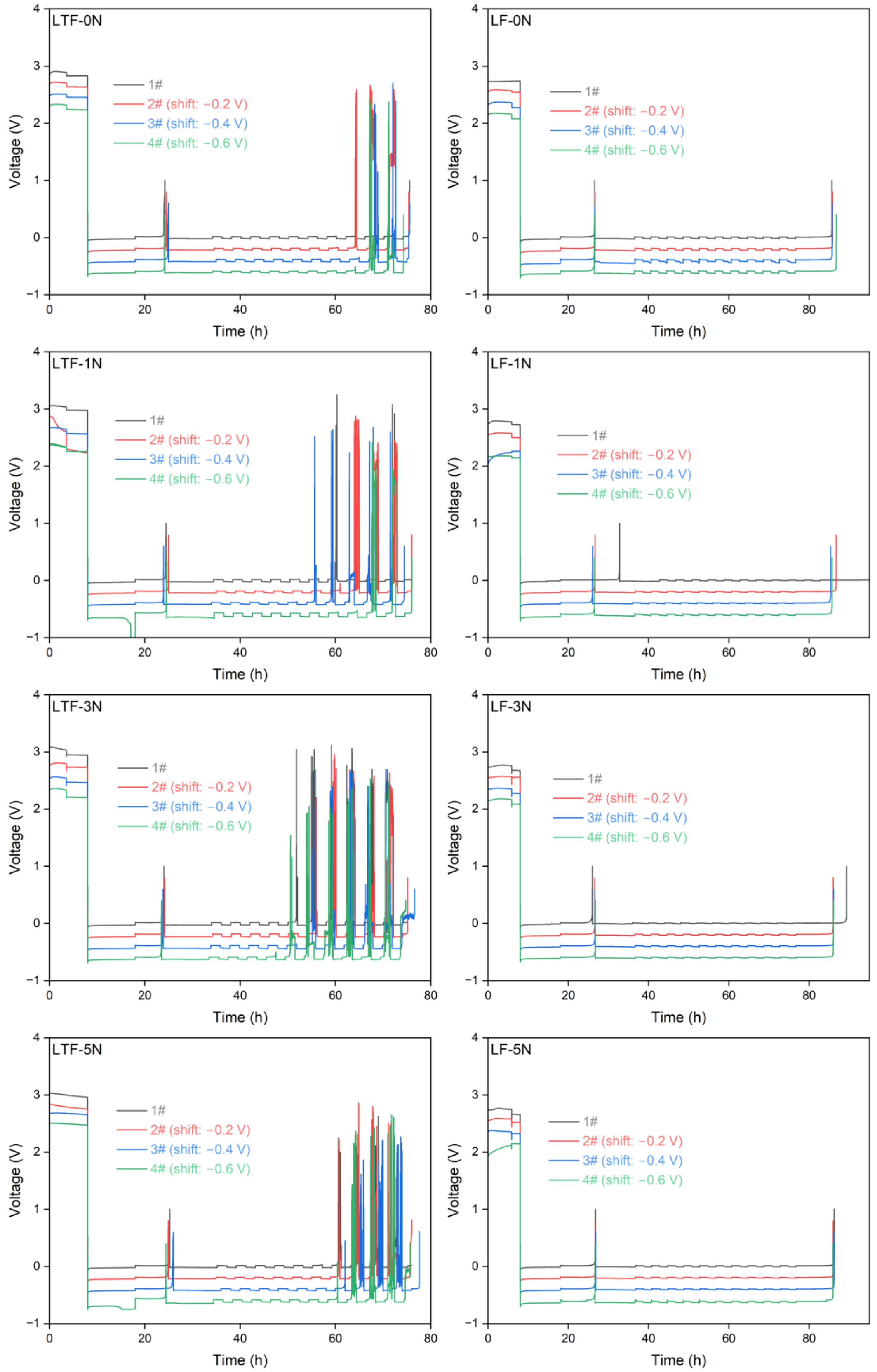

Figure S15. The source data of galvanostatic cycling profiles for multiple parallel samples.

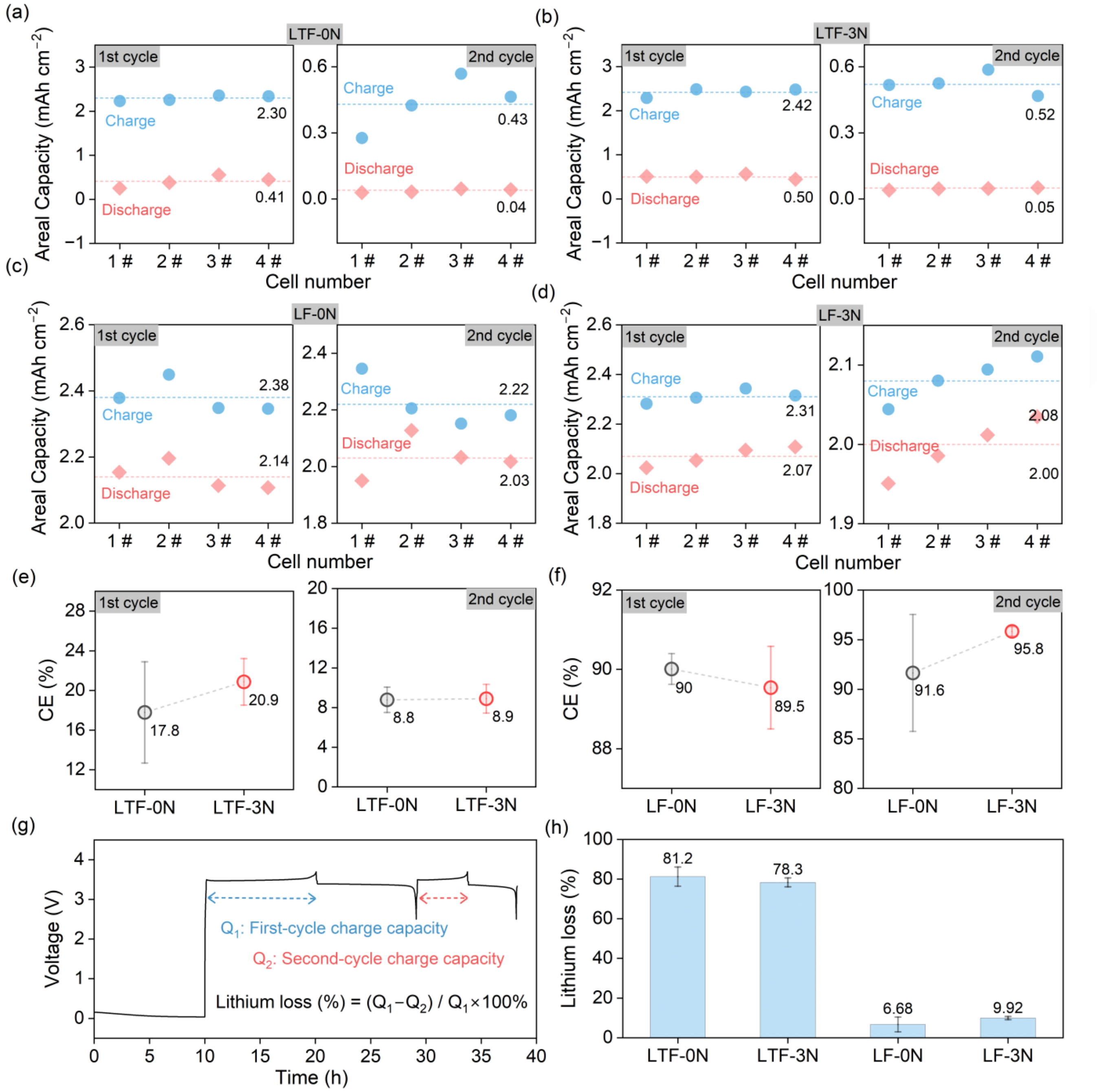

Figure S16. Electrochemical performance of the Cu°||$LiFePO_4$ cells with different electrolyte formulations. (a–d) First-cycle and second-cycle charge/discharge capacities of four parallel cells employing LTF-0N (a), LTF-3N (b), LF-0N (c), and LF-3N (d) electrolytes. Values shown in each panel correspond to the average capacities of the four cells. (e,f) Average first and second-cycle Coulombic efficiencies with standard deviation error bars for LiTFSI-based (e) and LiFSI-based (f) electrolytes. (g) Schematic illustration of active lithium loss quantification after two cycles of cycling. (h) Average active lithium loss with standard deviation error bars for LiTFSI-based and LiFSI-based electrolyte. Error bars represent the standard deviation obtained from four independent cells.

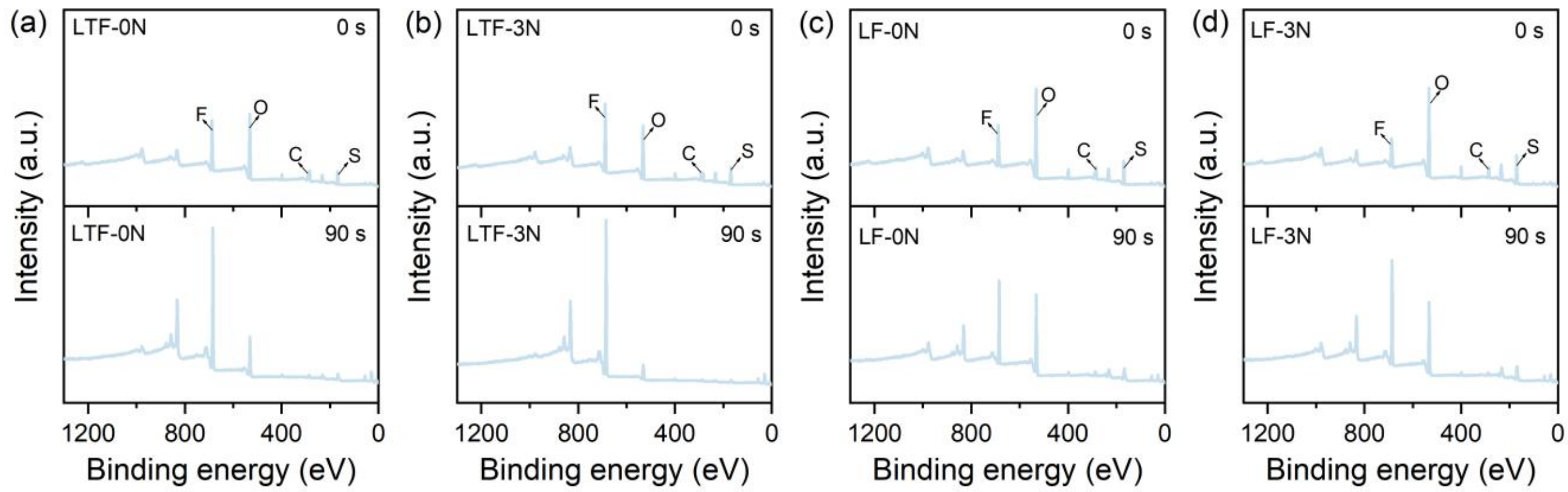

Figure S17. The XPS survey spectra of the Li° deposits recovered from LiTFSI-based (a and b) and LiFSI-based (c and d) electrolytes under the non-sputtered (0 s) and 90 s sputtered states.

WILEY-VCH

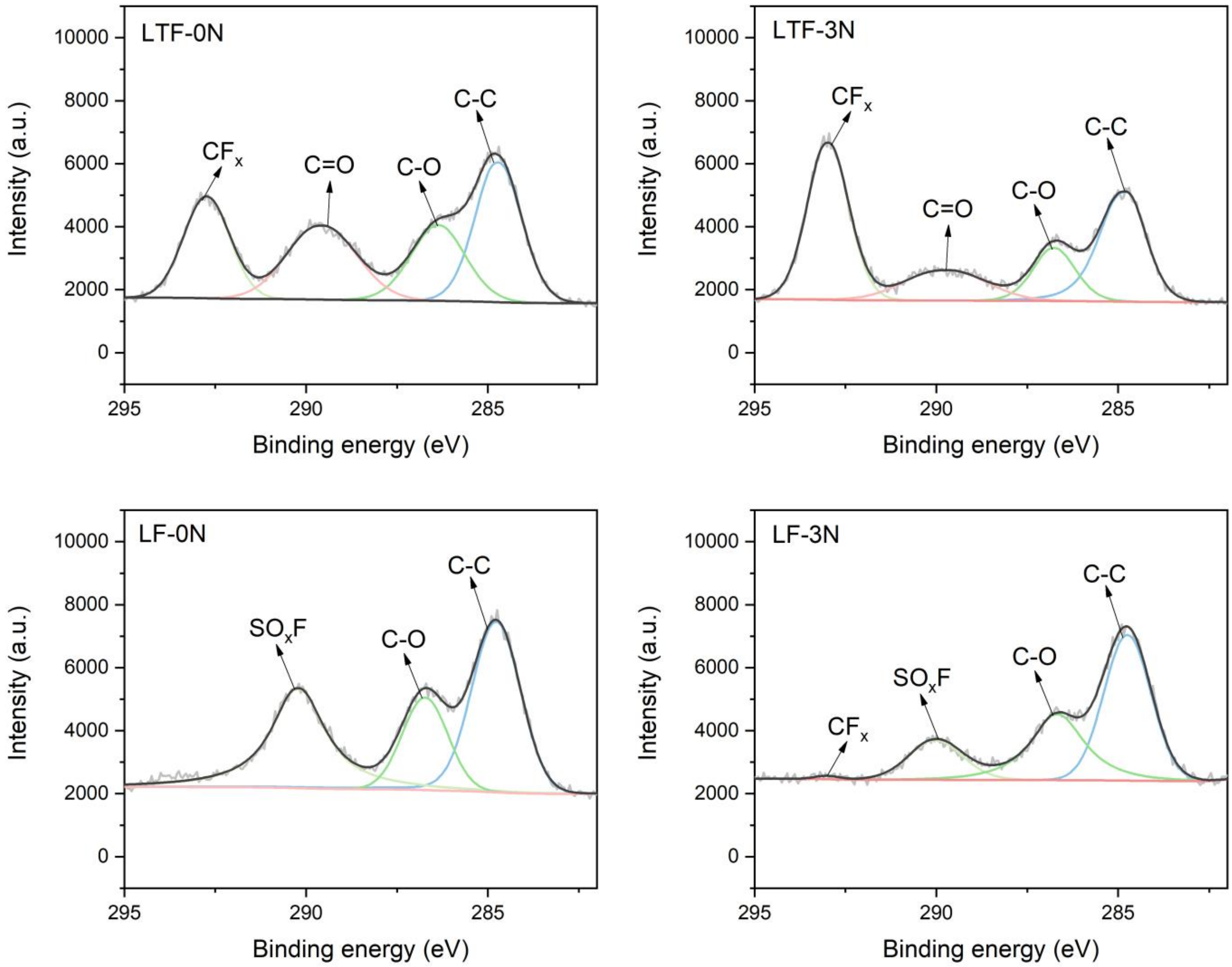

Figure S18. High-resolution C 1s XPS spectra of LiTFSI-and LiFSI-based electrolytes under the non-sputtered state.

WILEY

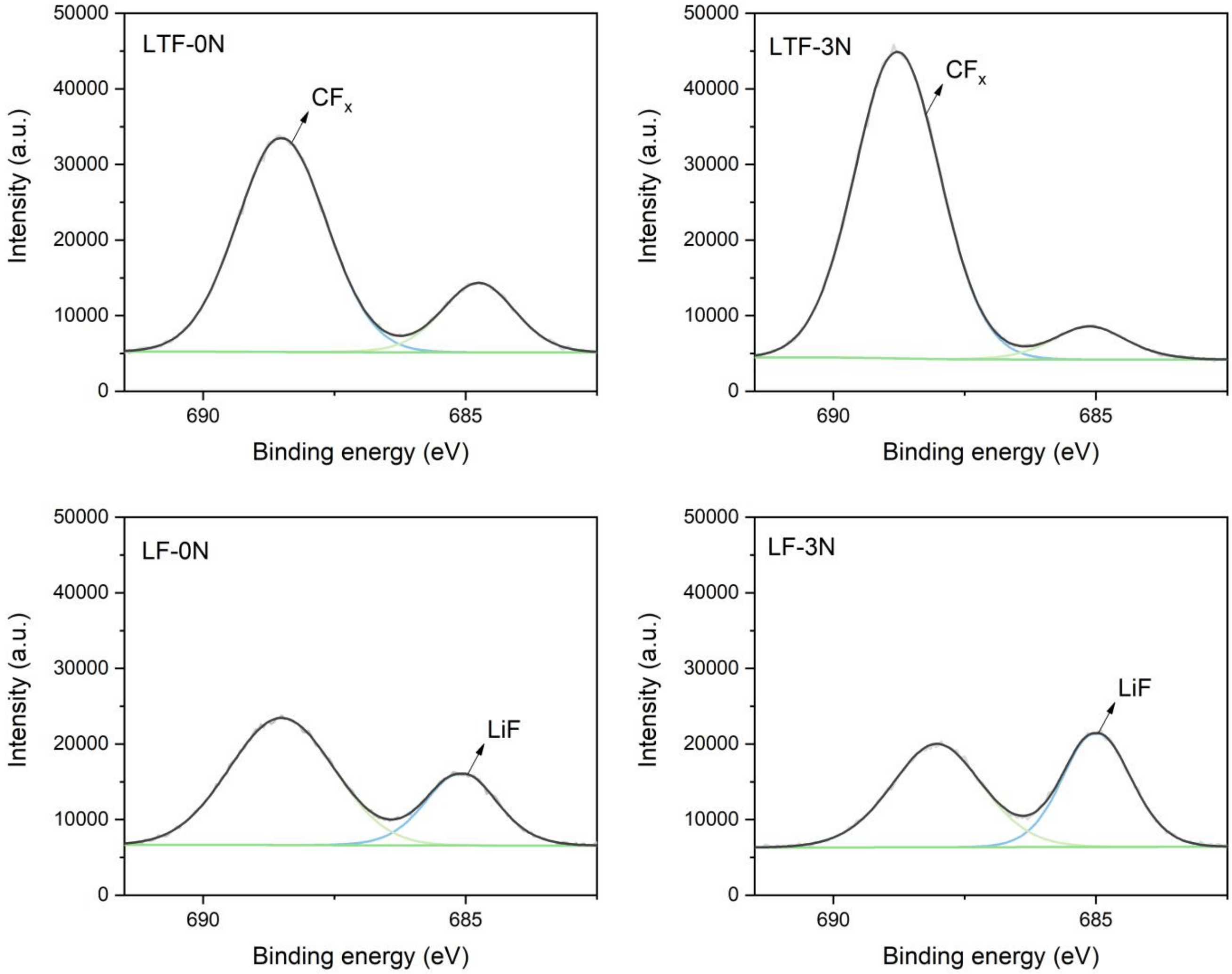

Figure S19. High-resolution F 1s spectra of LiTFSI and LiFSI-based electrolytes under the non-sputtered state.

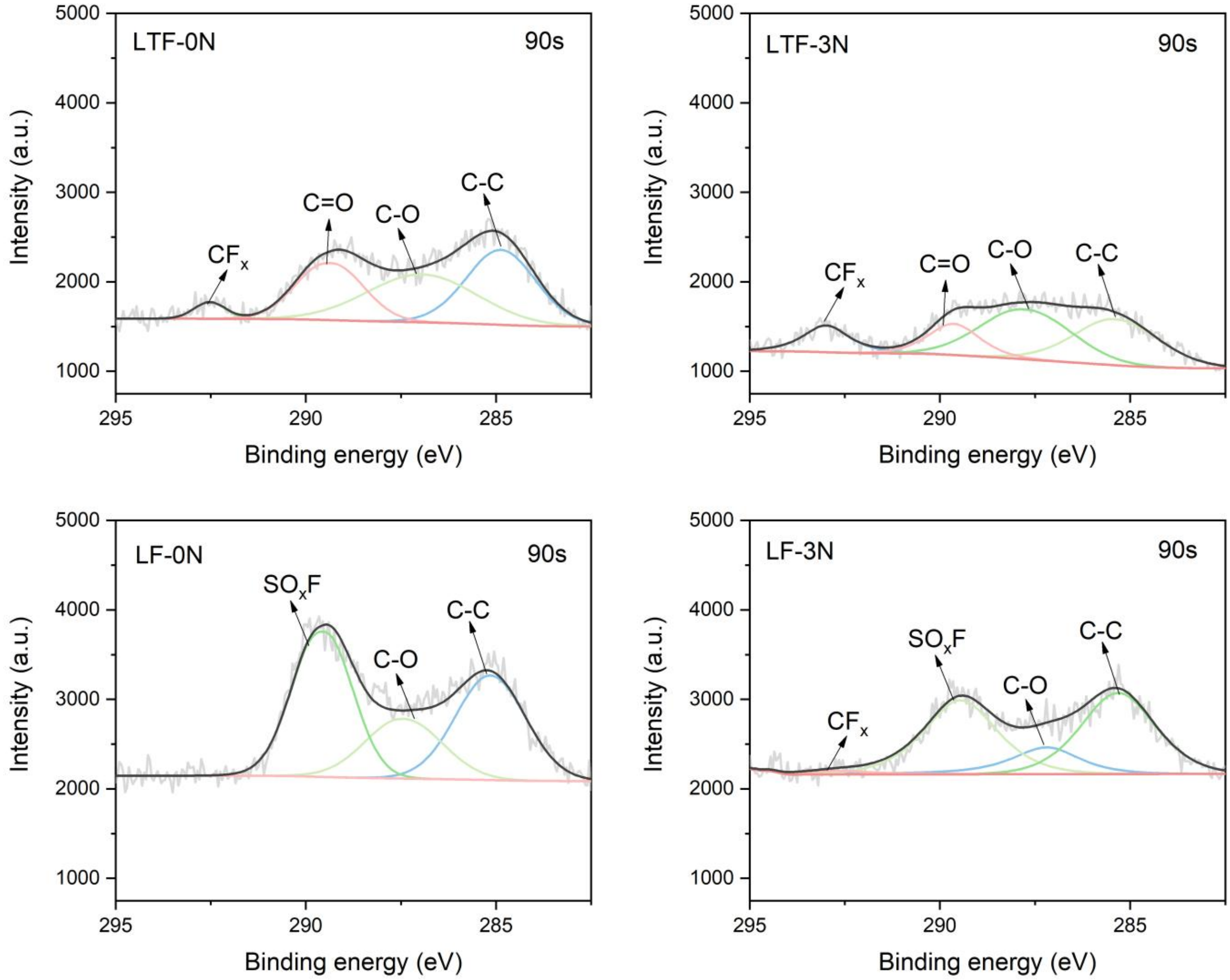

Figure S20. High-resolution C 1s spectra of LiTFSI and LiFSI-based electrolytes after 90 s sputtering.

WILEY

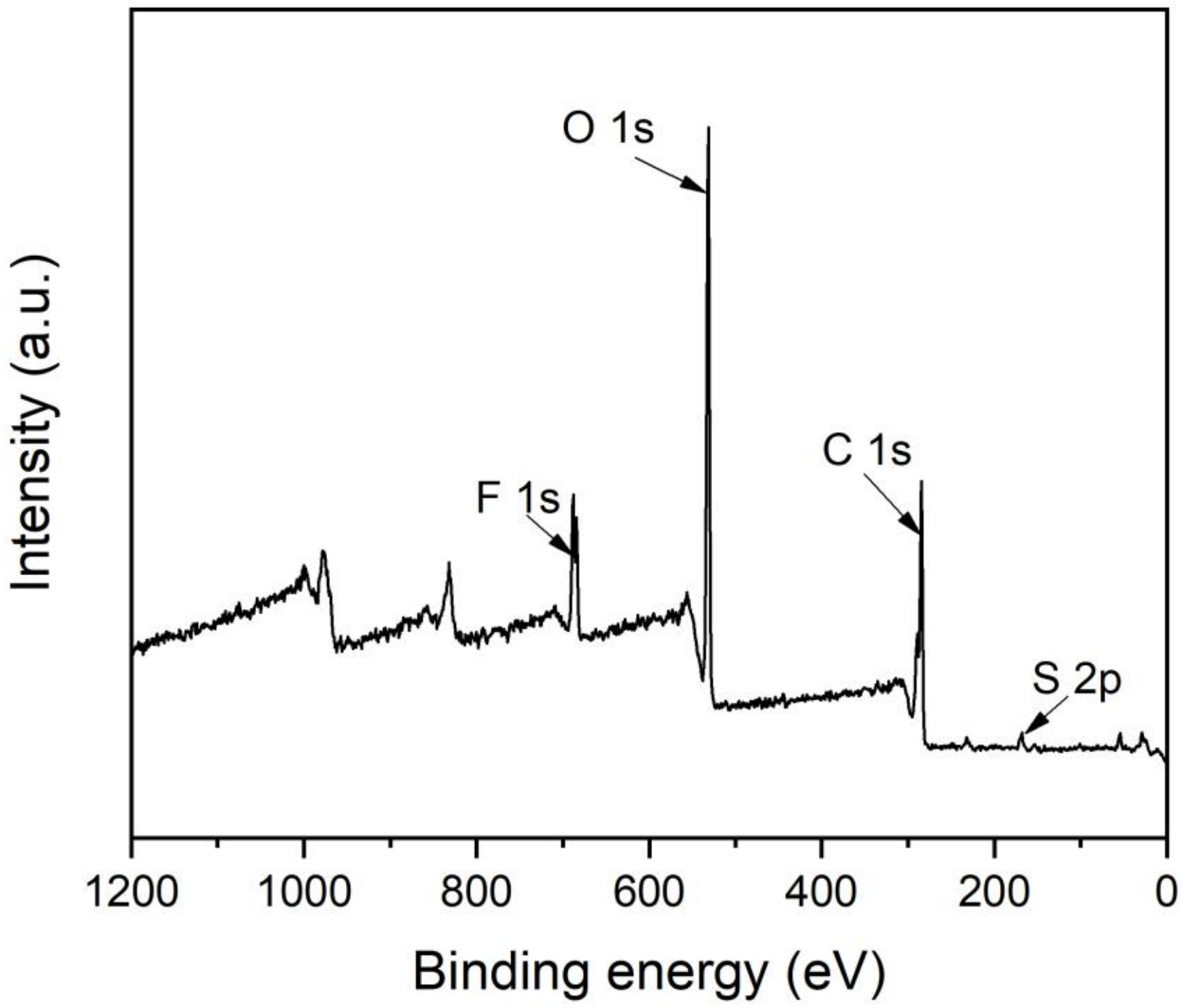

Figure S21. The XPS survey spectra of the solid phase in chemical simulation.

WILEY-VCH

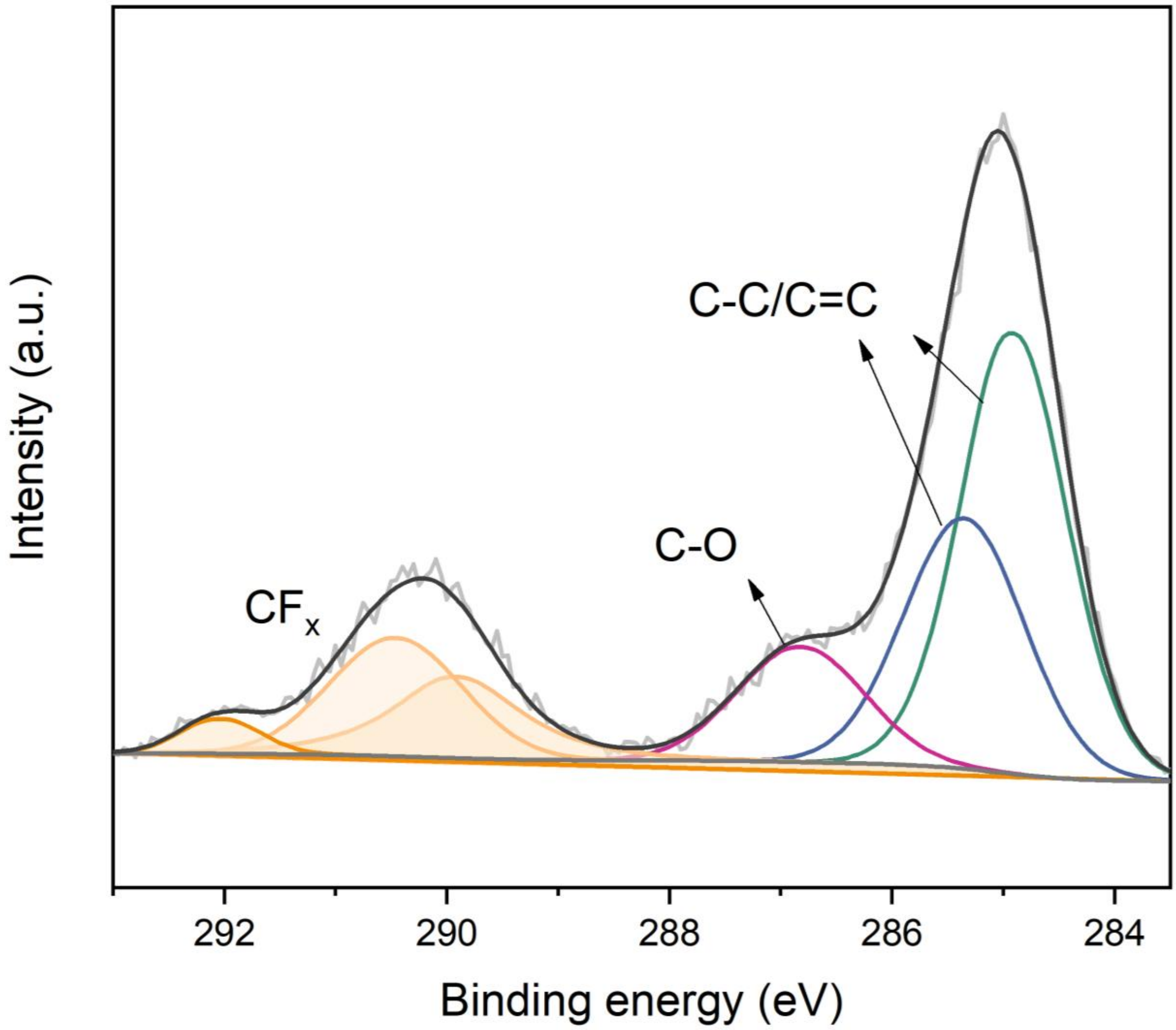

Figure S22. High-resolution C 1s XPS spectra of the solid phase in chemical simulation.

WILEY

References

[1] D. D. Perrin, W. L. F. Armarego, Purification of Laboratory Chemicals, 3rd ed., Pergamon Press, Oxford, **1988**.

[2] Z. Song, M. Tian, J. Zhu, J. Chen, W. Feng, L. Ben, H. Yu, X. Huang, M. Armand, Z. Zhou, H. Zhang, *Advanced Materials* **2024**, 36, 2410954.

[3] M. D. Hanwell, D. E. Curtis, D. C. Lonie, T. Vandermeersch, E. Zurek, G. R. Hutchison, *Journal of Cheminformatics* **2012**, 4, 17.

[4] V. Blum, R. Gehrke, F. Hanke, P. Havu, V. Havu, X. Ren, K. Reuter, M. Scheffler, *Computer Physics Communications* **2009**, 180, 2175–2196.

[5] A. D. Becke, *The Journal of Chemical Physics* **1993**, 98, 5648–5652.

[6] C. Lee, W. Yang, R. G. Parr, *Physical Review B* **1988**, 37, 785–789.

[7] F. Neese, *Wiley Interdisciplinary Reviews: Computational Molecular Science* **2022**, 12, e1606.

[8] J. G. Brandenburg, C. Bannwarth, A. Hansen, S. Grimme, *The Journal of Chemical Physics* **2018**, 148, 064104.

[9] S. Grimme, S. Ehrlich, L. Goerigk, *Journal of Computational Chemistry* **2011**, 32, 1456–1465.

[10] F. Weigend, R. Ahlrichs, *Physical Chemistry Chemical Physics* **2005**, 7, 3297–3305.

[11] A. Hellweg, D. Rappoport, *Physical Chemistry Chemical Physics* **2015**, 17, 1010–1017.

[12] S. Kossmann, F. Neese, *Journal of Chemical Theory and Computation* **2010**, 6, 2325–2338.

[13] T. Lu, F. Chen, *Journal of Computational Chemistry* **2012**, 33, 580–592.

[14] W. Humphrey, A. Dalke, K. Schulten, *Journal of Molecular Graphics* **1996**, 14, 33–38.

[15] E. D. Glendening, C. R. Landis, F. Weinhold, *Journal of Computational Chemistry* **2019**, 40, 2234–2241.

[16] M. J. Abraham, T. Murtola, R. Schulz, S. Páll, J. C. Smith, B. Hess, E. Lindahl, *SoftwareX* **2015**, 1–2, 19–25.

[17] J. N. Canongia Lopes, A. A. H. Pádua, *The Journal of Physical Chemistry B* **2004**, 108, 16893–16898.

[18] J. N. Canongia Lopes, A. A. H. Pádua, *Theoretical Chemistry Accounts* **2012**, 131, 1129.

[19] A. V. Marenich, S. V. Jerome, C. J. Cramer, D. G. Truhlar, *Journal of Chemical Theory and Computation* **2012**, 8, 527–541.

[20] L. Martínez, R. Andrade, E. G. Birgin, J. M. Martínez, *Journal of Computational Chemistry* **2009**, 30, 2157–2164.

[21] K. N. Wood, G. Teeter, *ACS Applied Energy Materials* **2018**, 1, 4493–4504.

[22] W. Guo, W. Zhang, Y. Si, D. Wang, Y. Fu, A. Manthiram, *Nature Communications* **2021**, 12, 3031.

[23] Y. Zheng, N. Yang, S. Duan, Z. Li, R. Gao, Y. Zhu, H. Wang, T. Zhang, G. Li, D. Luo, L. Yang, D. Wang, W. Yan, J. Zhang, Z. Chen, *Advanced Functional Materials* **2026**, 36, e11011.

[24] X. Liu, M. Zarrabeitia, A. Mariani, X. Gao, H. M. Schütz, S. Fang, T. Bizien, G. A. Elia, S. Passerini, *Small Methods* **2021**, 5, 2100168.

[25] S. Jiao, X. Ren, R. Cao, M. H. Engelhard, Y. Liu, D. Hu, D. Mei, J. Zheng, W. Zhao, Q. Li, N. Liu, B. D. Adams, C. Ma, J. Liu, J.-G. Zhang, W. Xu, *Nature Energy* **2018**, 3, 739–746.

[26] Z. Song, M. Tian, J. Zhu, J. Chen, W. Feng, L. Ben, H. Yu, X. Huang, M. Armand, Z. Zhou, H. Zhang, *Advanced Materials* **2024**, 36, 2410954.

WILEY-VCH

[27] Y. Wei, T. Liu, W. Zhou, H. Cheng, X. Liu, J. Kong, Y. Shen, H. Xu, Y. Huang, *Advanced Energy Materials* **2023**, 13, 2203547.
WILEY VCH
